# Immunofluorescence can assess the efficacy of mTOR pathway therapeutic agent Everolimus in breast cancer models

**DOI:** 10.1038/s41598-019-45319-4

**Published:** 2019-07-29

**Authors:** Chun-Ting Kuo, Chen-Lin Chen, Chih-Chi Li, Guan-Syuan Huang, Wei-Yuan Ma, Wei-Fan Hsu, Ching-Hung Lin, Yen-Shen Lu, Andrew M. Wo

**Affiliations:** 1 Institute of Applied Mechanics, National Taiwan University, Taipei, 106 Taiwan; 2 Department of Oncology, National Taiwan University Hospital, Taipei, 100 Taiwan

**Keywords:** Breast cancer, Cell signalling

## Abstract

When breast cancer patients start to exhibit resistance to hormonal therapy or chemotherapy, the mTOR inhibitor everolimus can be considered as an alternative therapeutic agent. Everolimus can deregulate the PI3K/AKT/mTOR pathway and affect a range of cellular functions. In some patients, the agent does not exhibit the desired efficacy and, even worse, not without the associated side effects. This study assessed the use of immunofluorescence (IF) as a modality to fill this unmet need of predicting the efficacy of everolimus prior to administration. Cell viability and MTT assays based on IF intensities of pho-4EBP1 Thr37/46 and pho-S6K1 Ser424 on breast cancer cells (Hs578T, MCF7, BT474, MDA-MB-231) and patient-derived cell culture from metastatic sites (ABC-82T and ABC-16TX1) were interrogated. Results show that independent pho-4EBP1 Thr37/46 and pho-S6K1 Ser424 IF expressions can classify data into different groups: everolimus sensitive and resistant. The combined IF baseline intensity of these proteins is predictive of the efficacy of everolimus, and their intensities change dynamically when cells are resistant to everolimus. Furthermore, mTOR resistance is not only consequence of the AKT/mTOR pathway but also through the LKB1 or MAPK/ERK pathway. The LKB1 and pho-GSK3β may also be potential predictive markers for everolimus.

## Introduction

The pathway PI3K/AKT/mTOR is frequently activated in breast cancer potentially controlling many major cytophysiologically functions related to cellular proliferation, metastasis, growth, survival and motility. When cells are stimulated by growth factors or hormones, phosphatidylinositol 3-kinase (PI3K) will likely be first activated promoting phosphorylation of both the AKT pathway and the mechanistic target of rapamycin complex 1 (mTORC1) sequentially. Then, the mTORC1 will phosphorylate its downstream substrate eukaryotic initiation 4E-binding protein 1 (4EBP1) and ribosomal protein S6 kinase beta-1 (S6K1)^1,2^. Moreover, p53, KRAS, PI3CA and PTEN mutations are well-known to be related to the PI3K/AKT/mTOR pathway^3–5^. As a consequence, the mammalian (or mechanistic) target of rapamycin (mTOR) pathway, in conjunction with phospho-4EBP1 and phospho-S6K1, plays a critical role in controlling key cellular processes^6–8^.

For breast cancer, mTOR-targeted therapy is considered when patients are resistant to hormonal therapy or chemotherapy. The therapeutic agent everolimus is one of mTOR inhibitors and can deregulate the PI3K/AKT/mTOR pathway by allosteric binding of the catalytic site of mTORC1. Anti-proliferative effect of everolimus has also been demonstrated in cancer cell lines^9^. Everolimus can be used combinatorically with other agents with efficacy in clinical trials. For example, a landmark study (BOLERO-2) for hormone receptor-positive and human epidermal receptor 2 (HER2)-negative advanced breast cancer patients demonstrated everolimus and exemestane extended progression-free survival (PFS) compared with exemestane and placebo, with median PFS of 10.6 months verses 4.1 months, respectively (hazard ratio, 0.36; 95% CI, 0.27 to 0.47; P < 0.001)^10^. A follow-up study explored the correlation of genetic alterations in a representative cohort of BOLERO-2 patients found that the efficacy of everolimus was essentially independent of the status of PIK3CA, FGFR1, and CCND1, which are the commonly altered genes or pathways associated with this cohort^11^.

In clinical trials, combination therapy was employed yet it was still difficult to confirm the effect or impact of each agent. To conquer this obstruction, many researchers had been working hard and several studies had modeled combinatorial therapeutic effect with algorithmic methods while considering the probability of sensitivity of each individual agent^12,13^. The expression of individual agent might give another way/method to predict/find potential sensitivity of combination treatments^14^. It demonstrates that everolimus monotherapy is still worth examining with *in vitro* and vivo to reveal mechanism of resistance to this drug in breast cancer. Instead of replacing the existing combined therapy, the core research value of this work is to find a more efficient way to understand and show the patient’s medication situation.Clinical consideration of everolimus when appropriate could benefit from a means to predict the response of the agent for patients prior to administration while weighting the potential side effects which could substantially hamper life quality for some patients. The predictive modality should be amenable to the clinical management of the disease with relative ease given the fact that these patients have been shown to be resistant to hormonal therapy or chemotherapy.

A range of methodologies have been explored to interrogate the mTOR pathway. Many reported responses downstream of the mTOR pathway using western blot on cell lines^15–19^ and immunohistochemistry (IHC) on tumors^19–21^. While others have directly targeted the mTOR pathway and a range of inhibitors using quantitative PCR (q-PCR), IHC and western blot to demonstrate the efficacy of these mTOR inhibitors^18–24^. Clinically, IHC is routinely used to characterize tumor tissue, while western blot and immunofluorescence (IF) are research tools for protein studies. Each modality is robust when utilized within its designed parameters, which is often balanced by complex considerations such as amount of sample required, processing time, clinical setting and cost. Some studies reported gene expression and mutations can be leveraged to assess the sensitivity of everolimus^15,24–29^, yet another research revealed that low chromosome instability corresponds to everolimus efficacy, while no difference in PIK3CA mutation status^11^. Another study also hypothesized that efficacy of the agent is related to epithelial-mesenchymal transition (EMT) and cell lines were systematically interrogated^30^ towards this end.

Although many methodologies have been undertaken on everolimus, further studies to explore the use of IF as a modality to predict response of the therapeutic agent are still warranted for the following reasons: (a) the IHC approach is challenging particularly when phospho-antibodies are involved; (b) even if western blot and IHC might detect protein expressions for mTOR pathway, the results might be challenging to interpret due to rapid dephosphorylation of some phosphorylated protein during the duration required^31^; (c) IF can be detected in media for fresh or frozen samples to avoid dephosphorylation of phosphorylated protein; and (d) IF had been proved to correlate linearly with results from western blot^32^ while, in contrast, IF enables rapid processing, requires less sample than other protein detection approaches which is important in the metastatic setting where samples can be limited if accessible. Key characteristics among IHC, western blot and IF are listed in Table 1 ^31,33–35^.

**Table 1:** Comparsions between western blot, IHC and IF used in the study^31,33–35^.

	Western blot	Immunohistochemistry	Immunofluorescence
*Target*	Protein expressions	Protein expressions	Protein expressions
*Sample acquisition*	Proteins lysated form cells or tissues	Tissues	Cells or tissues (supension or adherent cells)
*Sample quantity*	cells typically (about 15–30 μg total proteins)	cells from tissues typically	cells typically (1/10–1/100 times compared with amount of western blot)
*Advantages*	Sensitivite detection	Can measure situations in the context of intact tissues.	Can be used in suspension and adherent cells situations
	Quantitative analysis use		Can be used in fresh samples situations
	Provides moleualr weight information		Less sample need amount and less time to perform results
			Quantitative analysis
			Suitable for detection for phosho-proteins
*Disadvantages*	Time consuming problem	Less sensitivity quantitatively	The condition setting for high sensitivity
	High sample comsumption	Limited time of storage	Limited time of storage
	Lower sensitivity in less protein amount situation	Repeated use problem	Repeated use problem
	Improper quantification		
	Measuring confusion when phosphorlyative peotein detection		
	Reactivity problem of antibodies each other		

Besides, it could be presumed that pho-4EBP1 and pho-S6K1 are not only due to AKT/mTOR pathway but activated by another target or pathway from some studies, which suggested that pho-GSK3β and LKB1 would affect the downstream of mTOR pathways^36,37^. Supplementary Fig. 1 showed the possible factors that affected the performance of pho-4EBP1 and pho-S6K1. This study was also focused on expressions of pho-4EBP1 and pho-S6K1.

The aim of this study was to interrogate IF as a predictive modality for the response of the mTOR inhibitor everolimus monotherapy in breast cancer cell lines and in patient-derived cell culture (PDCC) from metastatic breast cancer sites. The reason for the focus of everolimus monotherapy is that, as aforementioned, the mTOR pathway inhibitor is considered not only for endocrine-resistance but also for chemo-resistance patients. Thus, unlike other combinatorial study where the complementary agent contributes in a supporting therapeutic role, it is reasonable to assume that the addition of endocrine or chemo therapeutics should not contribute substantially to determine multi-agent sensitivity/resistance in everolimus-based therapy. This study design serves as a pilot attempt to develop a clinically amenable tool towards the goal set in this work.

## Materials and Methods

### Cell culture, cell lines, and patient-derived cell culture (PDCC)

Breast cancer cell lines MCF7, MDA-MB-231, BT474, Hs578T and two PDCC ABC-16TX1, ABC-82T were chosen to explore the use of IF signals to predict the sensitivity of the mTOR inhibitor everolimus. All experiments were tested in triplicate.

MCF7 cell lines were cultured in 10% FBS DMEM/F12 medium. BT474 cell lines were cultured in Hybricare medium, which EGF was added. MDA-MB-231 cell lines were cultured in 10% FBS DMEM HG medium. Hs578T cell lines were cultured in 10%FBS DMEM HG medium, which insulin was added. Patient-derived cell culture (PDCC) ABC82T and ABC-16TX1 were unique cells, which were derived from breast cancer patient metastatic biopsy samples, and these two PDCC were cultured in IH medium. All cells were incubated in the culture dish (704001, NEST) in 37 deg. C, 5% CO_2_ incubator. Supplementary Table 1 and supplementary Table 2 list the characteristics and the gene mutation of these cell lines and PDCC^25,38,39^.

### Reagents

The following reagents and kits were used: cell fixation buffer and Alexa Fluor 488 anti-human CD24 antibody (BioLegend), cell permeabilization kit (MiltenyiBiotec), mouse anti-human CD45 conjugated with Alexa Fluor 488 and Hoechst33342 (Invitrogen), Pho-4EBP1 Thr37/46 antibody, vimentin antibody conjugated with Alexa Fluor 647 and pho-GSK3β Ser9 antibody (Cell Signaling Technology), mouse anti-human CD44 antibody (BD Biosciences), Pho-S6K1 Ser424 antibody, pan cytokeratin C11 antibody, the donkey anti-rabbit and anti-mouse IgG H&L conjugated with Alexa Fluor 488 and 647 secondary antibodies (Abcam), pan cytokeratin AE1/AE3 antibody (Novus Biologicals), LKB1 antibody, goat anti-rabbit and anti-mouse IgG(H + L) superclonal secondary antibodies, HRP conjugated (Thermo Fisher), everolimus (Selleckchem), 10X TGS buffer and 10X TG buffer for western blot (Omics Bio), and MTT powder (Sigma-Aldrich).

### Cells viability rate assay

Cell lines MCF7, MDA-MB-231, BT474, and two PDCCs ABC-82T and ABC-16TX1 were used in this study. First, seeded cells in the 3.5 cm culture dish were grown to about 50–60% confluence. Second, 200 nM everolimus were added into the dish. After about 24 hours, cells were harvested and the number of live cells were counted and to determine cell viability rate. Cells under the normal growth condition were harvested as the control group. The cell viability rate is defined as equation (1):

1 $$\frac{{\rm{c}}{\rm{e}}{\rm{l}}{\rm{l}}\,{\rm{n}}{\rm{u}}{\rm{m}}{\rm{b}}{\rm{e}}{\rm{r}}\,{\rm{o}}{\rm{f}}\,{\rm{d}}{\rm{r}}{\rm{u}}{\rm{g}}\,{\rm{t}}{\rm{r}}{\rm{e}}{\rm{a}}{\rm{t}}{\rm{m}}{\rm{e}}{\rm{n}}{\rm{t}}\,{\rm{a}}{\rm{f}}{\rm{t}}{\rm{e}}{\rm{r}}\,{\rm{a}}{\rm{b}}{\rm{o}}{\rm{u}}{\rm{t}}\,24\,{\rm{h}}{\rm{o}}{\rm{u}}{\rm{r}}{\rm{s}}-{\rm{c}}{\rm{e}}{\rm{l}}{\rm{l}}\,{\rm{n}}{\rm{u}}{\rm{m}}{\rm{b}}{\rm{e}}{\rm{r}}\,{\rm{o}}{\rm{f}}\,{\rm{c}}{\rm{o}}{\rm{n}}{\rm{t}}{\rm{r}}{\rm{o}}{\rm{l}}\,{\rm{a}}{\rm{f}}{\rm{t}}{\rm{e}}{\rm{r}}\,{\rm{a}}{\rm{b}}{\rm{o}}{\rm{u}}{\rm{t}}\,24\,{\rm{h}}{\rm{o}}{\rm{u}}{\rm{r}}{\rm{s}}}{{\rm{c}}{\rm{e}}{\rm{l}}{\rm{l}}\,{\rm{n}}{\rm{u}}{\rm{m}}{\rm{b}}{\rm{e}}{\rm{r}}\,{\rm{o}}{\rm{f}}\,{\rm{c}}{\rm{o}}{\rm{n}}{\rm{t}}{\rm{r}}{\rm{o}}{\rm{l}}\,{\rm{a}}{\rm{f}}{\rm{t}}{\rm{e}}{\rm{r}}\,{\rm{a}}{\rm{b}}{\rm{o}}{\rm{u}}{\rm{t}}\,24\,{\rm{h}}{\rm{o}}{\rm{u}}{\rm{r}}{\rm{s}}}\times 100{\rm{ \% }}$$

### MTT and IC50 assays

About 5,000–10,000 cells were seeded in 100 μl medium into the 96-well and incubated in 37 C at CO_2_ 5% incubator overnight before the drug treatment. Second, the same volume of the culture medium which contained different everolimus concentration were replaced and were incubated for 48 hours.

After treatment with everolimus for 48 hours, the culture medium was replaced by 12 mM 100 μl MTT solution and was placed for 3 hours in 37 C at CO_2_ 5% incubator. Finally, the same amount of DMSO was replaced for 20 minutes at 37 C and the absorbance of DMSO at 570 nm was determined, which stood for the number of live cells by the plate reader (MQX 200, BioTek). The concentration of IC50 to everolimus were determined for every type of cell lines.

### Immunofluorescence staining

For IF staining, cells were harvested first, then fixation buffer 200 μl was added and incubated for 20 minutes at 4 C and washed with running buffer. Second, cells were permeabilized by adding the inside perm buffer, incubated for 20 minutes at 4 C and washed with running buffer (some antibodies did not use this process).

After cells were fixed and/or permeabilized, the primary antibodies were added, incubated for 25 minutes at 4 C and washed with running buffer. When primary antibodies were bonded at cells, the secondary antibodies conjugated to Alexa Fluor 488 or 647 channels were added, incubated for 25 minutes at 4 C and washed with running buffer. Immunofluorescence images were taken by microscope.

### Western blots

The following protocol was used for western blot. Cells of about 10^6^ count were boiled with sample buffer for 15 minutes. Afterwards, cell samples which contained proteins were loaded into the wells of gels (Mini-PROTEAN Precast Gels; Bio-Rad). Then, the gels were run with 200 volts for about 30 minutes. Upon completion, proteins were transferred to PDVF membranes (Merck Millpore) at 100 volts for 30 minutes.

After running gels and blotting, the PDVF membranes were stained with Ponceau S solution for about 5 minutes to show the total proteins expression to check the equal amount protein and were washed with PBS-T three times. After this, the PDVF membranes were blocked with milk at room temperature for about 2 hours. After blocking, the primary antibody (1:1000) were added into the milk, incubated at 4 C overnight and were washed with PBS-T three times. The secondary antibody which HRP conjugated (1:5000) was added into the milk, incubated at room temperature for 1 hours and washed with PBS-T six times. Proteins were detected using imaging (UVP BioSpectrum 500 Imaging System).

### Statistical analysis

Immunofluorescence images in this work were captured (from Leica DMI 6000 B microscope) and analyzed by a commercial software (MetaMorph). In the software, the count nuclei function was used which can enumerate cells above a certain threshold of IF intensity. After image capture and counting, another software (SigmaPlot 12.5 and MedCalc Version 18.11.3) was used for compilation and results expressed as mean $\frac{+}{-}$ s.e.m.

## Results

### Cell viability and IC50 of cell lines & PDCC

Initial cell viability was first assessed prior to testing in the MTT assay. Cells – BT474, MCF7, MDA-MB-231, ABC-82T and ABC-16TX1 – were placed under 200 nM everolimus treatment alone for 24 hours and compared with the amount under the normal growth condition over the same time interval in a cell viability assay. Results are presented in Supplementary Fig. 2 showing the growth rate of BT474 and MCF7 cells are “negative” while growth of MDA-MB-231, ABC-82T and ABC-16TX1 are “positive” implying resistant to everolimus.

Table 2 and Supplementary Fig. 3 represent results for the MTT assay and IC50 values under different everolimus concentrations. Results show that the inhibition rate of Hs578T and MCF7 cells are less than 100 nM and inhibition rate of BT474 cells is about 60 nM, while results of MDA-MB-231, ABC-82T and ABC-16TX1 are still 70–80% under high everolimus concentrations. However, for ABC-16TX1 cells, the IC50 values were in the range of 0–10 μM of everolimus which suggest the cells are highly resistance to everolimus. Thus, we defined Hs578T and MCF7 cells as everolimus-sensitive cells and BT474, MDA-MB-231, ABC-82T and ABC-16TX1 cells as everolimus-resistant cells.

**Table 2:** The response to everolimus alone for cell lines and PDCC used in the study.

Cell lines and PDCC	IC50 concentration to everolimus	Respone to everolimus
*MCF7*	5 nM	Sensitive
*Hs578T*	6.8 nM	Sensitive
*BT474*	60 nM	Resistant
*MDA-MB-231*	500 nM	Resistant
*ABC-82T*	>500 nM	Resistant
*ABC-16TX1*	>1000 nM	Resistant

Notes: Sensitive to everolimus is defined as IC50 concentration < 50 nM

Resistance

to everolimus is defined as IC50 concentration > 50 nM.

### Immunofluorescence expression of mTOR markers and everolimus sensitivity

**Figure 1:**
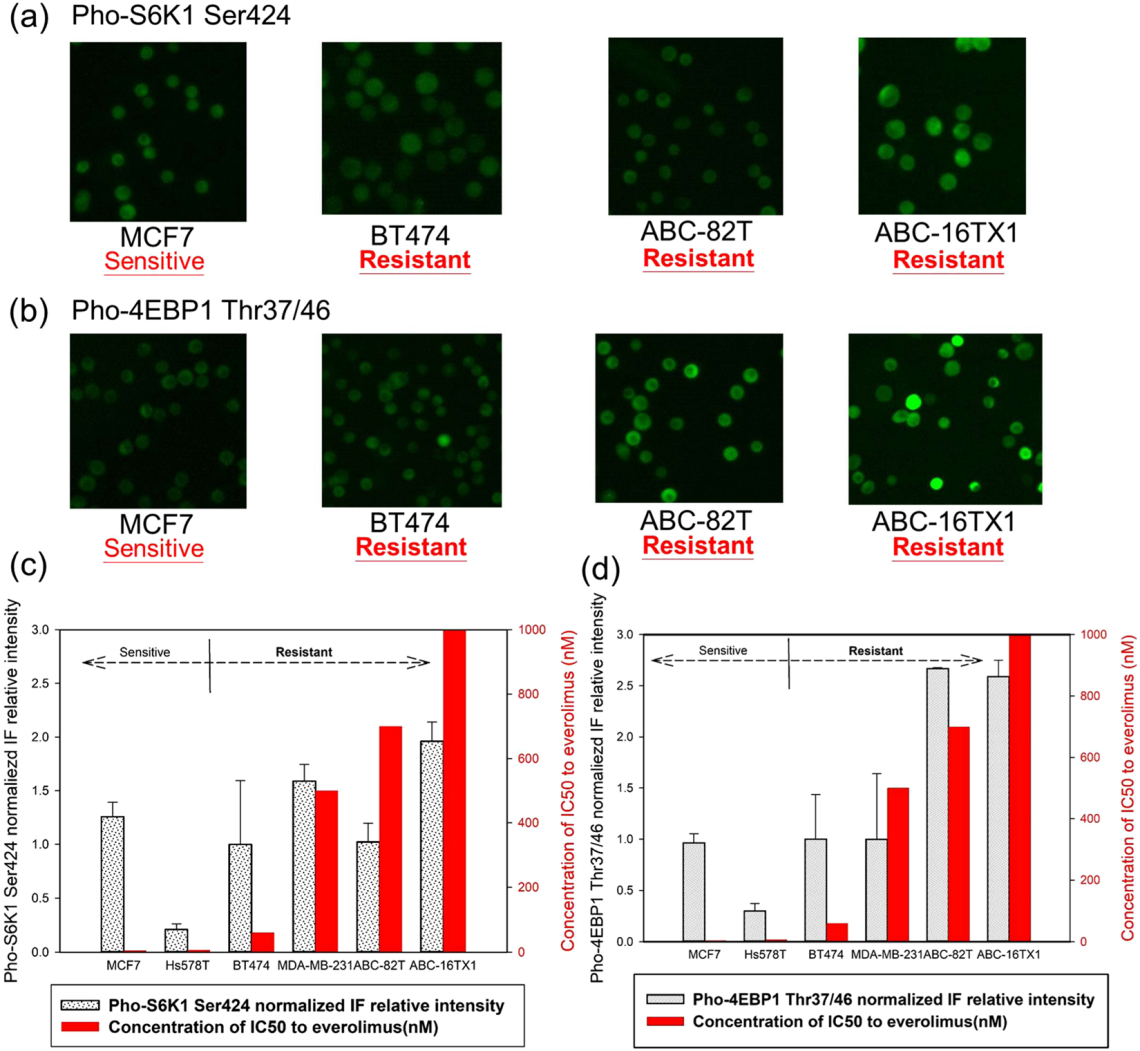
IF expression for BT474, MCF7, ABC-82T, and ABC-16TX1 cells without everolimus with (**a**) pho-S6K1 Ser424 and (**b**) pho-4EBP1 Thr37/46. Normalized IF intensity (using BT474) for (**c**) pho-S6K1 Ser424 and (**d**) pho-4EBP1 The37/46.

Based on the above assessment, Fig. 1 presents the baseline IF intensity for the same set of cells prior to administration of everolimus. Figure 1(a,b) show the images for the markers pho-S6K1 Ser424 and pho-4EBP1 Thr37/46, respectively. Analyses of these images are quantified in Fig. 1(c,d) with IF intensity normalized for the two antibodies (the intensity of BT474 cells for each antibody was used as normalization factor). In light of the results in Supplementary Figs 2 and 3 where sensitivity and resistance of each cell type are known, data in Fig. 1 suggest variation in IF intensity among these cells for both protein markers.

Results from Fig. 1(c,d) can be divided into four groups. The first group contains Hs578T and MCF7 cells, with both pho-S6K1 and pho-4EBP1 at low IF intensities demonstrating *sensitive* to everolimus. The rest of groups are everolimus *resistant*. The second group contains cells BT474 with similar pho-S6K1 and pho-4EBP1 IF intensities as the first group. The third group composes of (i) MDA-MB-231 cells with pho-S6K1 at high intensity and pho-4EBP1 low intensity and (ii) ABC-82T cells with pho-S6K1 at low intensity and pho-4EBP1 high intensity. The fourth group is ABC-16TX1 cells with both the pho-S6K1 and pho-4EBP1 IF signals at high intensities. These four groups are listed in Supplementary Table 3.

**Figure 2:**
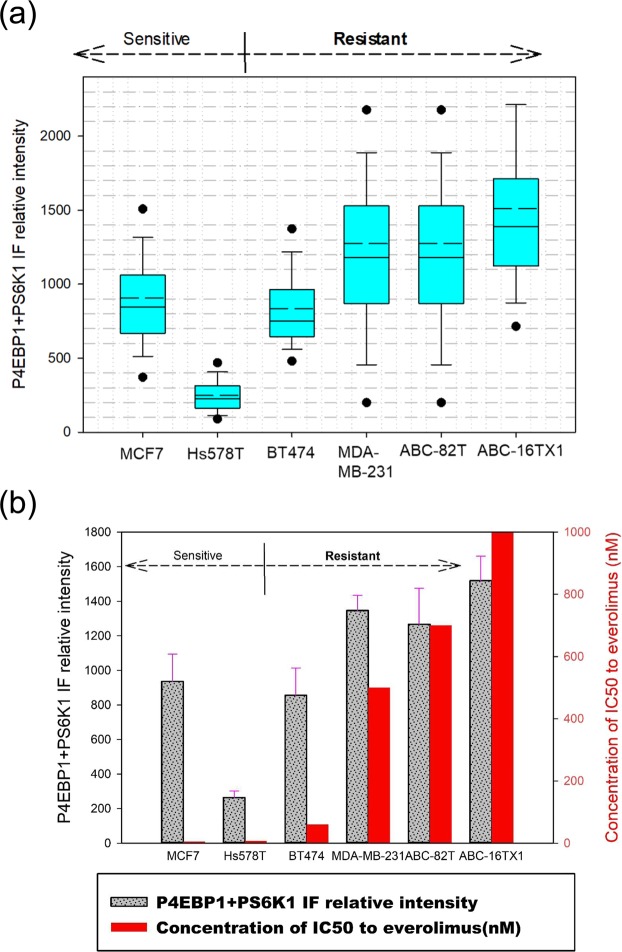
Antibodies-added (Combined) IF expression of pho-4EBP1 Thr37/46 and pho-S6K1 Ser424 for cell lines without everolimus. (**a**) Relative intensities of the two pho-antibodies. Everolimus-resistant cells have high IF intensity. (**b**) Comparison between IC50 values and the combined pho-antibody signals from (**a**).

Figure 2(a) and Supplementary Figure 4 present the combined IF images of pho-S6K1 and pho-4EBP1. In the context of this study, the manner in which the efficacy of everolimus is expressed is the key. From our experiment data, the individual use of either pho-4EBP1 or pho-S6K1 in immunofluorescence labeling might not better distinguish mTOR resistance intuitiveness than labeling both (combined) pho-4EBP1 and pho-S6K1 together. The resultant IF intensity from the combined labeling of the two antibodies clearly showed efficacy of administrating everolimus (see Supplementary Fig. 4 for double-staining of both antibodies using the same fluorescence channel).

Unlike Fig. 1(c,d) for individual marker, combined intensities suggest a clearer trend. Hs578T has the lowest relative intensity of all followed by MCF7 and BT474 with higher intensity. Hs578T and MCF7 cell lines are sensitive to everolimus therapy as demonstrated by results of Supplementary Figs 2 and 3. The cells MDA-MB-231, ABC-82T and ABC-16TX1 all have high relative intensity (relative to the background) and they are resistant to everolimus. Figure 2(b) represents the relationship between combined IF intensity and IC50 value. Results indicate that combined IF intensity is proportional to IC50 value in term of response to everolimus.

**Figure 3:**
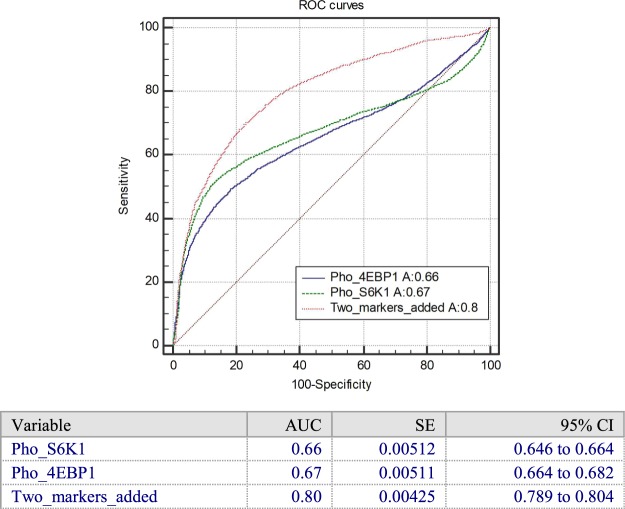
The ROC curve with pho-S6K1, pho-4EBP1 and the two added markers for everolimus sensitive/resistant cells group without everolimus. The combined IF intensity has a higher AUC than pho-4EBP1 and pho-S6K1 individually and can distinguish sensitive and resistant cells group statistically. (Amount of cells used in the test: 11092 cells, including six types of cell lines and PDCC. The parameter of ROC curves likes AUC, SE and 95% CI are listed).

Figure 3 and Supplementary Fig. 5(a) show the ROC curves and IF relative intensity for individual and combined protein markers for everolimus sensitive and resistant cell groups. The area of the ROC (AUC) for pho-4EBP1 only is 0.67 and the area of the ROC for pho-S6K1 only is 0.66. In comparison, the area of the ROC curve for *added* protein markers is 0.80. This AUC value suggests enhanced predictive power of everolimus sensitivity over that of pho-4EBP1 and pho-S6K1 individually.

### Pho-4EBP1 and pho-S6K1 reflect AKT/mTOR pathways

It is initially believed that the immunofluorescence of pho-4EBP1 and pho-S6K1 alone was sufficient to express the sensitivity of everolimus but the results of the experiment were not as we expected. These phosphorylated protein markers alone can only roughly cluster these cell lines, and cannot fully present the trend of everolimus resistance. Furthermore, in the original expectation, for cells that produce resistance in the mTOR drug everolimus, the amount of downstream phosphorylated protein should be expressed much more than those sensitive cells, as explained in the literature. However, from the experimental data of the individual immunofluorescence of phosphorylated proteins, it can be shown that MDA-MB-231 and ABC-82T cells do not fully comply with this argument. Therefore, it might be suggested that the performance of phosphorylated proteins downstream of mTOR is not entirely from the single AKT/mTOR pathway. Supplementary Fig. 1 indicates other physiological pathways or other proteins are also responsible for the performance of pho-4EBP1 and pho-S6K1^40,41^.

**Figure 4:**
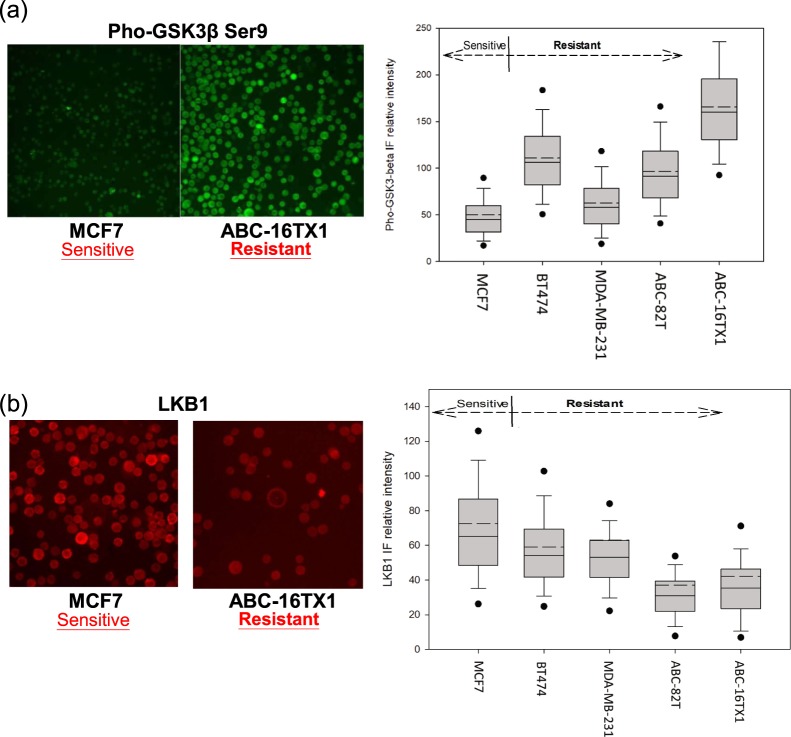
Pho-4EBP1 and pho-S6K1 reflect AKT/mTOR pathways. (**a**) IF relative intensity of pho-GSK3β. It suggests that the expression of pho-AKT cannot represent the resistance of everolimus. (**b**) Expressions and relative intensities of LKB1 for cell lines and PDCC without everolimus. Results show that everolimus-sensitive cells have higher LKB1 IF intensity than everolimus-resistant cells.

To test our hypothesis, another AKT downstream marker, pho-GSK3β Ser9 antibody, which represents activation of pho-AKT, and LKB1 antibody were chosen to interrogate whether IF intensity of pho-4EBP1 and pho-S6K1 would be impacted solely by AKT/mTOR. Figure 4(a) represents the IF relative intensity (FITC) for pho-GSK3β. Supplementary Fig. 5(b) show western blot for pho-GSK3β. The cells ABC-82T, ABC-16TX1 and BT474 cells have strong bands corresponding to pho-GSK3β at 46 kDa. In contrast, MDA-MB-231 and MCF7 cells have weak bands.

Figure 4(b) also shows the LKB1 IF signals (APC) for MCF7 and ABC-16TX1 cells, where Fig. 4(b) also quantifies intensities for all cell types studied. Supplementary Fig. 5(c) represents the western blot for LKB1. Everolimus sensitive cells MCF7 is higher than everolimus resistant cells ABC-16TX1^36,37^, means that LKB1 can affect everolimus efficacy.

### Antibodies-added IF expression of pho-4EBP1 and pho-S6K1 before and after everolimus

**Figure 5:**
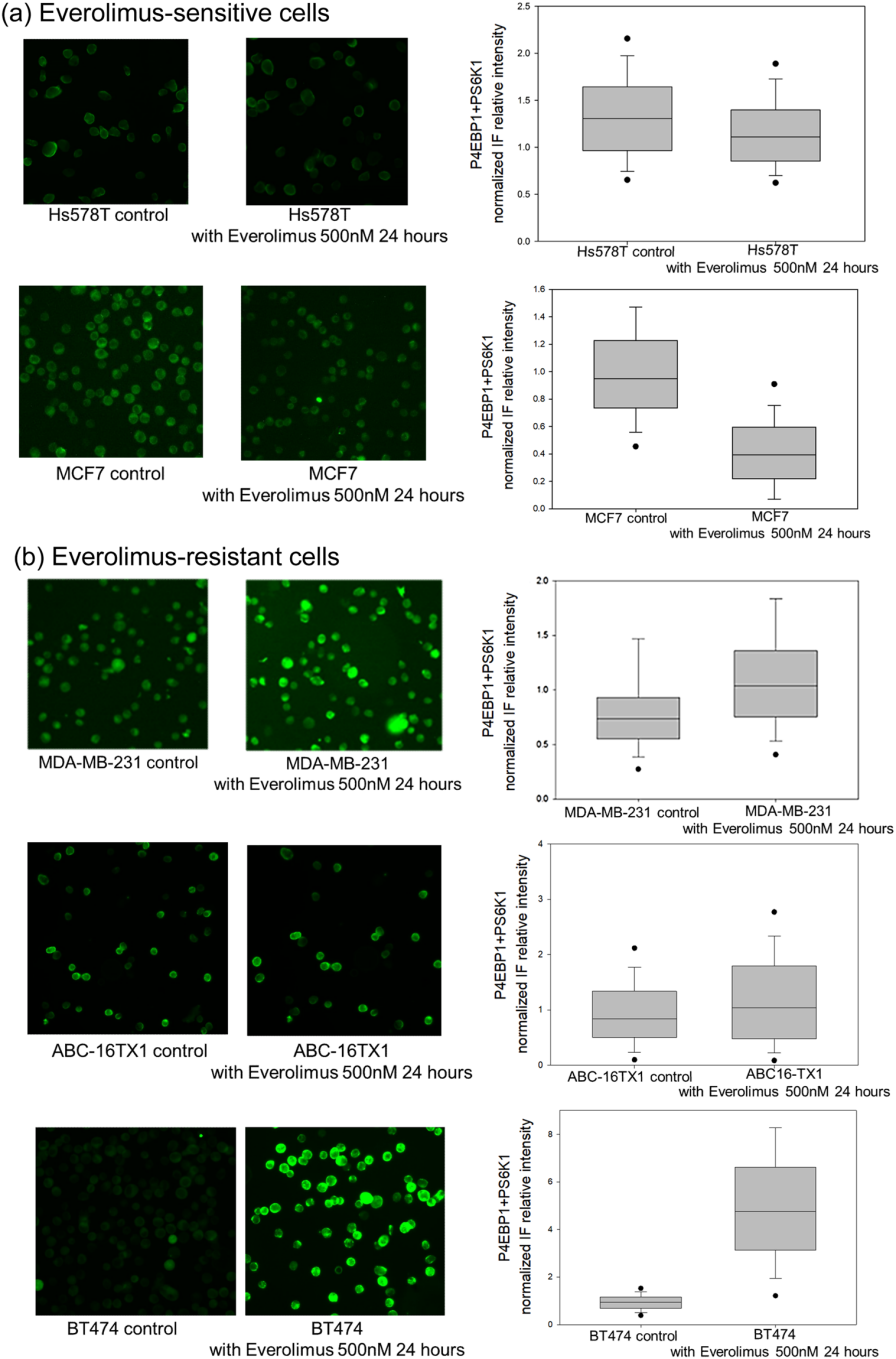
Antibodies-added (Combined) IF expression of pho-4EBP1 and pho-S6K1 for both the control group and the 500 nM everolimus group after 24 hours. (**a**) Everolimus-sensitive cells (Hs578T, MCF7 cell lines). (**b**) Everolimus-resistant cells (MDA-MB-231, BT474 cell lines and ABC-16TX1 PDCC).

With the aforementioned understanding, administration of everolimus was commenced. Figure 5 shows the combined expression of pho-4EBP1 and pho-S6K1 for everolimus-sensitive cells (Hs578T, MCF7) and everolimus-resistant cells (BT474, MDA-MB-231, ABC-16TX1) for the control group (without everolimus) and with application of everolimus at 500 nM after 24 hours. Figure 5(a) shows the IF signals of MCF7 and Hs578T decreased after everolimus addition but the signals of everolimus-resistant cells remained essentially unchanged. The BT474 cell line in everolimus 500 nM group are higher than the control group, and this cell line also has higher resistance than MCF7 cell line. Figure 5(b) shows that BT474, MDA-MB-231 cells and ABC-16TX1 cells are everolimus-resistant cells.

### Epithelial-mesenchymal transition state and everolimus sensitivity

**Figure 6:**
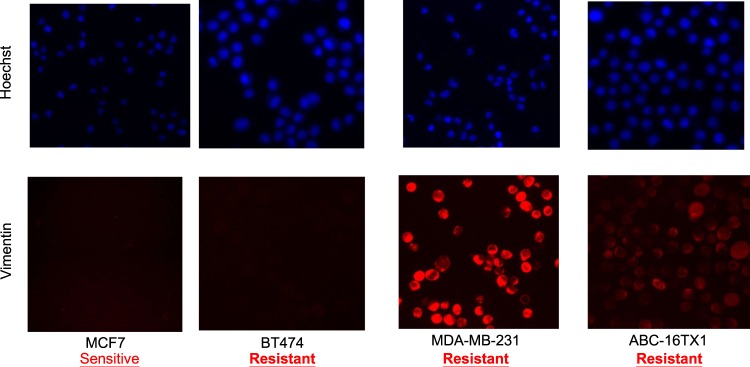
IF expression of vimentin for cell lines and PDCC (red: vimentin; blue: nucleus) for everolimus sensitive and resistant cells. Results suggest likely relationship between EMT state and sensitivity of everolimus where vimentin positive is indicative of everolimus resistant cells.

The efficacy of everolimus is reportedly affected by the cellular program epithelial-mesenchymal transition (EMT)^30^. We used an EMT marker (vimentin) to interrogate this understanding. Figure 6 presents results for four cell types. The expression of vimentin is negative for MCF7 which is sensitive to everolimus. On the other hand, the expression is positive for MDA-MB-231 and ABC-16TX1 which are resistant to everolimus. These results provide clue to the positive correlation between EMT state and the efficacy of everolimus.

## Discussion

The main goal of this study was to assess the use of IF as a modality to predict the efficacy of the mTOR inhibitor everolimus alone in breast cancer cells. Fundamentally, the mTOR pathway inhibitor everolimus is considered not only for endocrine-resistance but also for chemo-resistance patients. Furthermore, mTOR therapy with everolimus might be not completely straightforward to confirm a correlation with original resistance of each cell type to everolimus alone. In general, combinatorial therapy might bring more clinical benefits than monotherapy. The reference^42^ demonstrated that combination therapy offers PFS benefits. However, it also clearly demonstrated that monotherapy offers OS benefits in the same reference. Hence, it is preferred not to make a clear-cut statement in this paper that combination therapy offers definitive clinical benefits. Thus, as aforementioned, unlike other combinatorial study where the complementary agent contributes in a supporting therapeutic role, the way of applications of drugs maybe not the dominant factor or its affected factors is flexible to determine everolimus resistance. It is reasonable to assume that the addition of endocrine or chemo therapeutics or protein inhibition should not contribute totally to determine dominant everolimus sensitivity/resistance.

The high expression of pho-S6K1 and pho-4EBP1 in some cells may reflect mutation of mTOR. Two references^43,44^ also provided evidence that the expression of mTOR downstream marker will increase when the mTOR genes are mutated. Further studies suggested that high expression of pho-4EBP1 Thr37/46 due to resistant to rapamycin^11,45^. Moreover, pho-S6K1 Ser424 is also related to the MAPK/ERK pathway, which is another pathway in cell proliferation. In a gastric cell line research, expressions of pho-S6K1 Ser424 and pho-AKT Ser473 in rapamycin-resistant cells were higher than that in rapamycin-sensitive cells^46^. Another research also showed that low pho-S6 expression corresponded to response to everolimus-combined therapy and pho-S6K1 overexpression was associated with a poor prognosis^47^.

Our results (Fig. 4, Supplementary Figs 5(b,c), 6 and 8) suggest the expression of pho-AKT cannot represent the resistance of everolimus. One reference^40^ also indicated that the higher expression of pho-GSK3 in lung cancer cell lines, the higher the resistance of everolimus. The results presented here confirm this phenomenon and show that the mTOR resistance might be impacted not only by the AKT/mTOR but also by LKB1 or another pathway, e.g. the MAPK/ERK pathwa**y**. Our results may also be shown the same expressions of pho-GSK3β and negative feedback of pho-AKT. If cells have characteristics of resistance in everolimus-sensitive cells, the dynamic change for pho-4EBP1 and pho-S6K1 will commence. For everolimus-resistant cells, the expression of pho-4EBP1 and pho-S6K1 will almost unchanged. These results also show that signals are proper in our research.

Results presented suggest that IF as a modality can predict the efficiency of everolimus as a monotherapy in the current models. The use of IF can substantially reduce the time required compared with western blot. Besides, IF can potentially be applied even in rare cells when minimal samples are available, while methods of western blot, q-PCR and IHC would need a certain amount of samples to show expressions and quantifications of target protein. Moreover, the dephosphorylative level of phosphorylated protein markers in mTOR pathway will also be relative with saving conditions of samples and experiment time. If saving condition of samples is not proper or total experiment time is too long, it might lead to a misunderstanding result. Based on accuracy of phosphorylated protein considerations, IF might provide a better choice to investigate biomarkers of mTOR researches.

Collectively, data suggest that IF intensities of combined pho-4EBP1 Thr37/46 and pho-S6K1 Ser424 markers can predict everolimus sensitivity in the cell models and metastatic cell culture used. It also indicates that mTOR resistance might be impacted not only by AKT/mTOR but also by LKB1 or MAPK/ERK pathway. In addition, the expression of LKB1 and pho-GSK3β may also be potential markers for efficacy of the therapeutic agent.

## Supplementary information

Supplementary Information
