# Author Correction: Immunofluorescence can assess the efficacy of mTOR pathway therapeutic agent Everolimus in breast cancer models

**DOI:** 10.1038/s41598-020-68553-7

**Published:** 2020-08-19

**Authors:** Chun-Ting Kuo, Chen-Lin Chen, Chih-Chi Li, Guan-Syuan Huang, Wei-Yuan Ma, Wei-Fan Hsu, Ching-Hung Lin, Yen-Shen Lu, Andrew M. Wo

**Affiliations:** 1 Institute of Applied Mechanics, National Taiwan University, Taipei, 106 Taiwan; 2 Department of Oncology, National Taiwan University Hospital, Taipei, 100 Taiwan

Correction to: *Scientific Reports* 10.1038/s41598-019-45319-4, published online 29 July 2019

This Article contains errors.

As a result of a Figure assembly error, in Figure 5B the image for ABC-16TX1 control is a duplicate of the image for ABC-16TX1 with Everolimus. The correct Figure 5 is included below as Figure 1.

**Figure 1:**
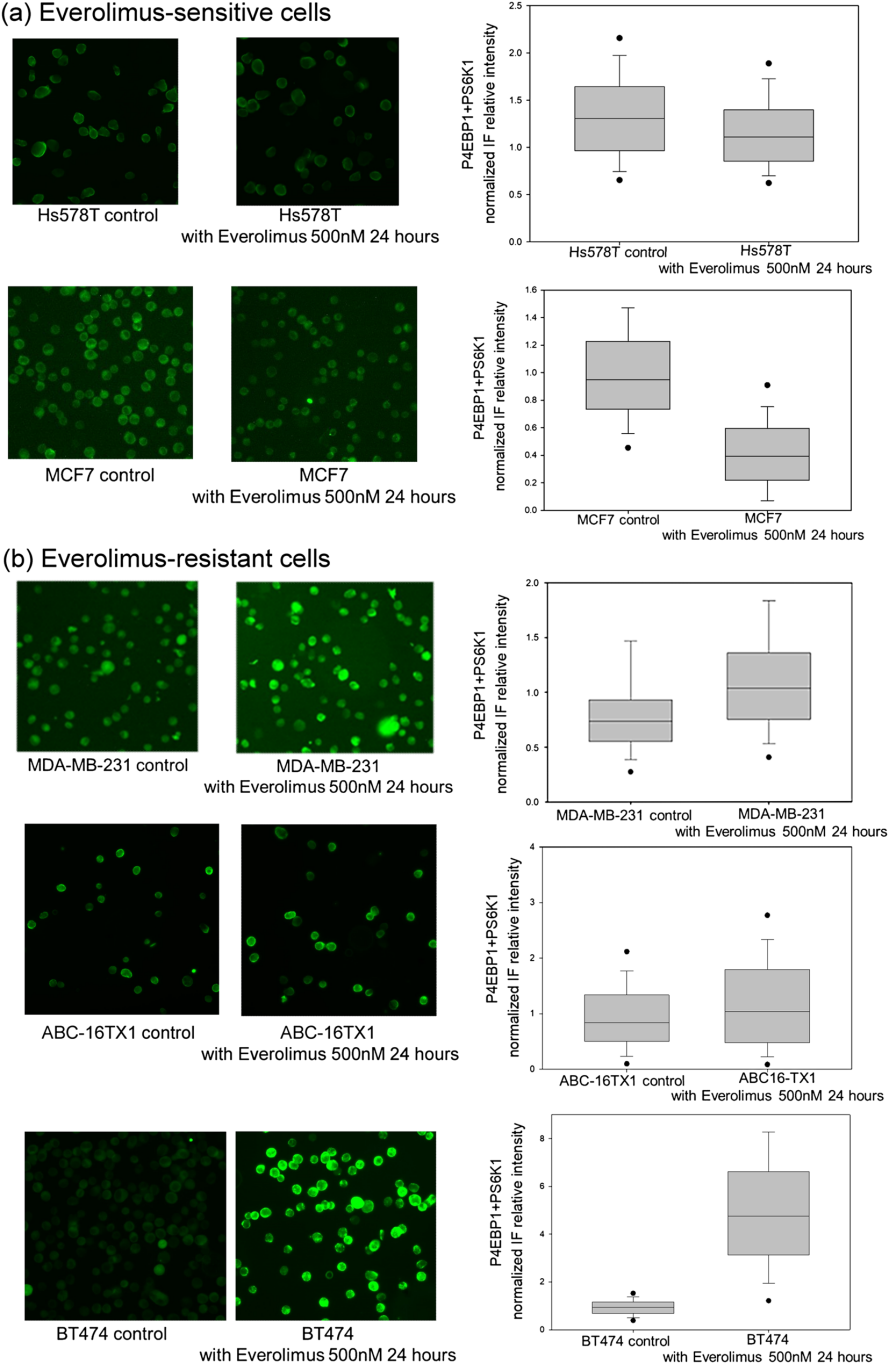
Antibodies-added (Combined) IF expression of pho-4EBP1 and pho-S6K1 for both the control group and the 500 nM everolimus group after 24 hours. (**a**) Everolimus-sensitive cells (Hs578T, MCF7 cell lines). (**b**) Everolimus-resistant cells (MDA-MB-231, BT474 cell lines and ABC-16TX1 PDCC).

The legend of Supplementary Figure 2, “Cell proliferation situations under the 200 nM everolimus treatment alone after 24 hours.”

Should read:

“Cell proliferation situations under the 200 nM everolimus treatment alone after about 24 hours.”

The legend of Supplementary Figure 4 “The resultant IF intensity from the combined labeling of the two antibodies clearly showed efficacy of administrating everolimus.”

Should read:

“The resultant IF intensities from the combined labeling of the two antibodies clearly show efficacy of administrating everolimus.”

The legend of Supplementary Figure 5 “(b) Western blot expression with ponceau S solution showing the total protein. The expression of black band at 46 kDa in western blot represents the amount of pho-GSK3β protein. (c) Western bolt expression of LKB1 for cell lines. The expression of black band at 54 kDa in western blot represents the amount of target protein.”

Should read:

“(b) Western blot expressions with ponceau S solution showing the total protein. The expressions of black band at 46 kDa in western blot represent the amount of pho-GSK3β protein. (c) Western bolt expressions of LKB1 for cell lines. The expressions of black band at 54 kDa in western blot represents the amount of target protein.”

The legend of Supplementary Figure 6 “The full-length western blot expression for pho-GSK3β with ponceau s solution, which shows the total protein. The expression of black band at 46 kda in western blot stand for amount of targeted protein.”

Should read:

“The full-length western blot expressions for pho-GSK3β with ponceau s solution, which shows the total protein. The expressions of black band at 46 kda in western blot stand for amount of targeted protein.”

The legend of Supplementary Figure 7 “The full-length western blot expressions for pho-GSK3β with beta-actin as loading control. The expression of black band at 46 kda in western blot stand for amount of pho-GSK3β, and the expressions of the black band at 48 kda represent amount of loading control.”

Should read:

“The full-length western blot expressions for pho-GSK3β with beta-actin as loading control. The expressions of black band at 46 kda in western blot stand for amount of pho-GSK3β, and the expressions of the black band at 48 kda represent amount of loading control.”

The legend of Supplementary Figure 8 “The full-length western blot expression for LKB1 for cell lines with ponceau s solution, which shows the total protein. The expression of black band at 54 kda in western blot stand for amount of targeted protein.”

Should read:

“The full-length western blot expressions for LKB1 for cell lines with ponceau s solution, which shows the total protein. The expressions of black band at 54 kda in western blot stand for amount of targeted protein.”

In Supplementary Table 1, the heading “Gene mutation for cell lines and PDCC used in the study [15,35-36]”

Should read:

“Gene mutation for cell lines and PDCC used in the study [25,38-39]”

In Supplementary Table 3, the heading “The group of cells by pho-S6K1 and pho-4EBP1 in the study”

Should read:

“Four groups of cells by pho-S6K1 and pho-4EBP1 IF intensities in the study”

Additionally, in Supplementary Table 3, column one was relabelled “IF intensities” and moved to column three. An additional column one was added, labelled “Group”, and a typographical error in column four heading “Respone to everolimus from immunofluorescence” was adjusted.

The final column order of Supplementary Table 3

Should read:

Column one “Group”, column two “Cell lines and PDCC”, column three “IF intensities” and column four “Responses to everolimus from immunofluorescence”.

Finally, the following changes are in the corrected Supplementary Information file to improve readability:

Supplementary Figure 1, the layout of the flowchart.

Supplementary Figure 3, the colour and size of the internal graph labels “Sensitive cells” and “Resistant cells” were altered.

Should read:

“Sensitive” and “Resistant”

Supplementary Figure 4, the colour, size and wording of the image labels “Everolimus-sensitive cells” and “Everolimus-resistant cells” were altered.

Should read:

“Sensitive” and “Resistant”

Supplementary Figure 5, the size and spacing of the x-axis labels for image (a) were altered.

The corrected Supplementary Information file is linked to this correction notice. These changes do not affect the conclusions of the Article.

## Supplementary information

Supplementary information

