# Supplementary material for: Immunofluorescence can assess the efficacy of mTOR pathway therapeutic agent Everolimus in breast cancer models
Source: Sci Rep. 2019 Jul 29;9:10898. doi: 10.1038/s41598-019-45319-4 (PMC6662705; doi:10.1038/s41598-019-45319-4)
Supplement: Supplementary file 1 — Supplementary Information [file 41598_2019_45319_MOESM1_ESM.docx]

**Immunofluorescence can assess the efficacy of mTOR pathway therapeutic agent Everolimus in breast cancer models (Supplementary Information)**

Chun-Ting Kuo1,*, Chen-Lin Chen1,*, Chih-Chi Li1, Guan-Syuan Huang1, Wei-Yuan Ma1,

Wei-Fan Hsu1, Ching-Hung Lin2, Yen-Shen Lu2,***, Andrew M. Wo1,**

1 Institute of Applied Mechanics, National Taiwan University, Taipei 106, Taiwan

2 Department of Oncology, National Taiwan University Hospital, Taipei 100, Taiwan

* These authors contributed equally to this paper.

** Corresponding author, e-mail: [andrew@iam.ntu.edu.tw](mailto:andrew@iam.ntu.edu.tw), phone: 886-2-3366-5656

*** Corresponding author, e-mail: [yslu@ntu.edu.tw](mailto:yslu@ntu.edu.tw)


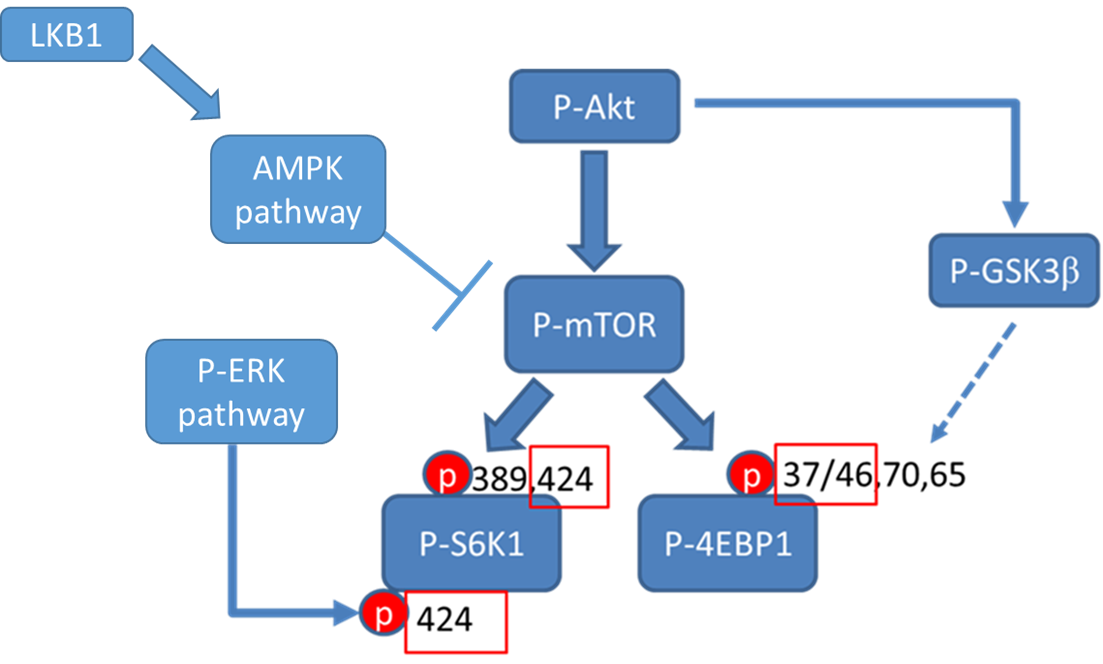


Supplementary Figure 1. Pho-4EBP1 and pho-S6K1 are not only due to AKT/mTOR pathway but activated by another target or pathway.


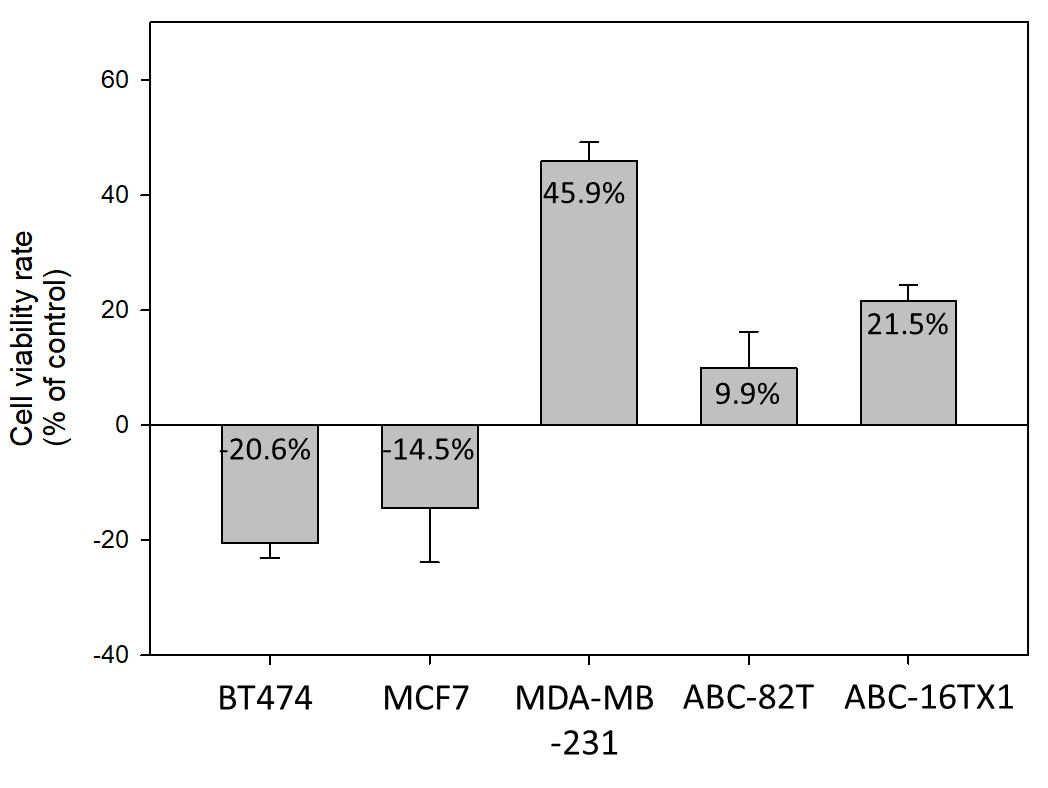


Supplementary Figure 2. Cell proliferation situations under the 200 nM everolimus treatment alone after 24 hours. Some cells were found as sensitive to everolimus (cell growth rate is negative) where the others are resistant to everolimus (cell growth rate is negative).

(BT474, MCF7, MDA-MB-231, ABC-82T and ABC-16TX1 cells are chosen.)


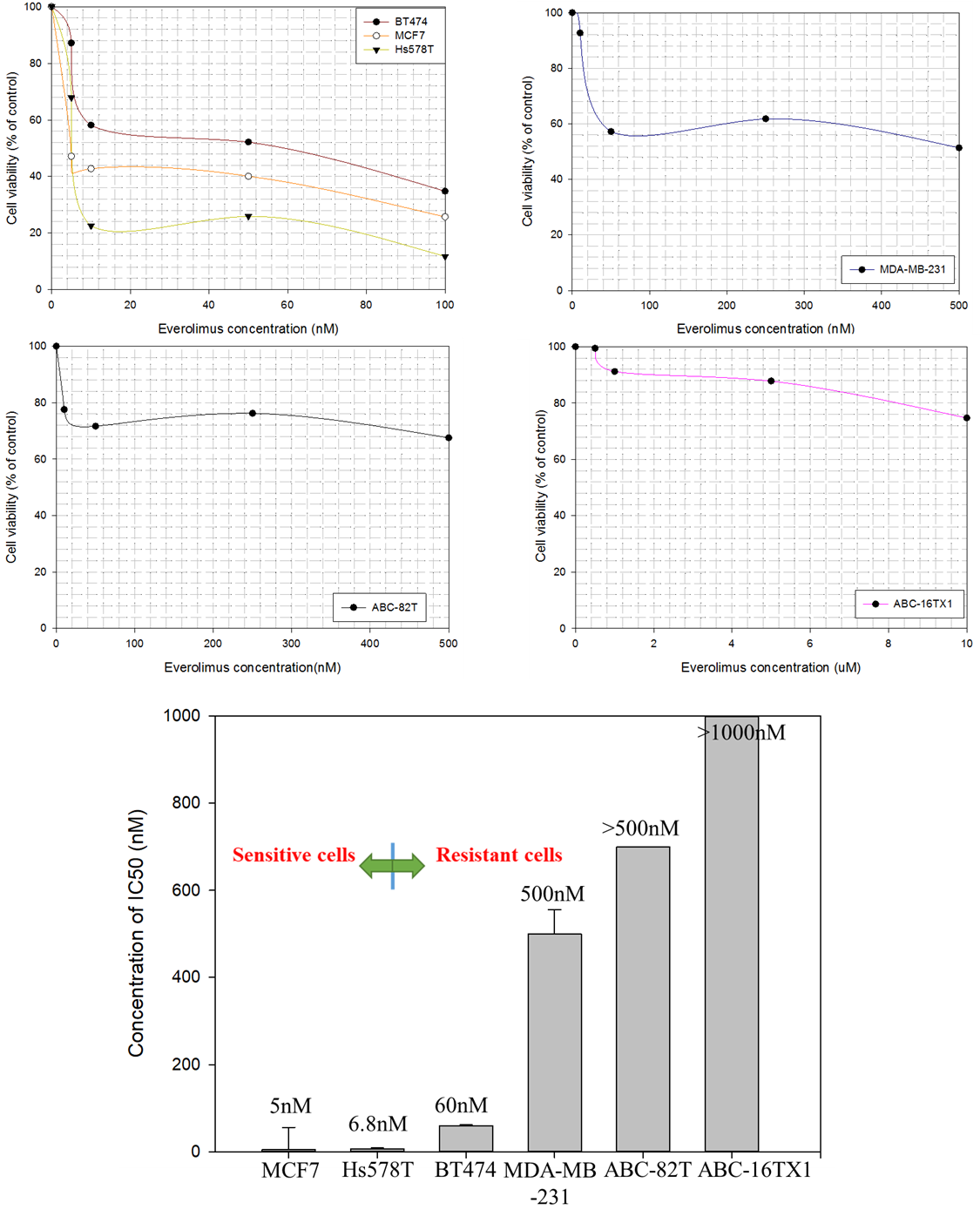


Supplementary Figure 3. Cell survival curve and IC50 concentration to everolimus for cell lines and PDCC by MTT assay.

(Hs578T, BT474, MCF7, MDA-MB-231, ABC-82T and ABC-16TX1 cells are chosen.)


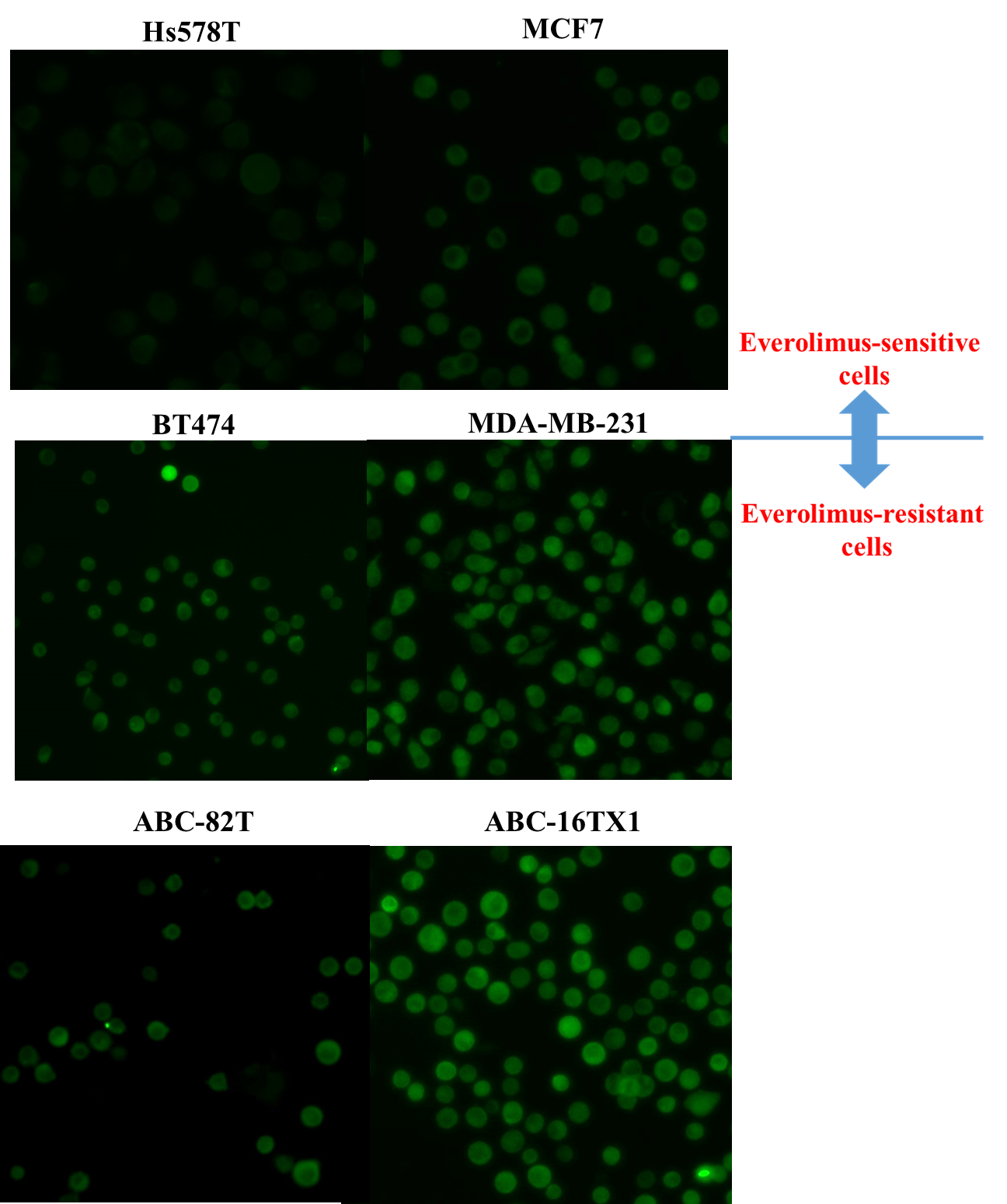


Supplementary Figure 4. Combined IF expressions in six type’s breast cancer cell lines and PDCC. From the experiment data, the individual use of either pho-4EBP1 or pho-S6K1 in immunofluorescence labeling might not better distinguish mTOR resistance intuitiveness than labeling both (combined) pho-4EBP1 and pho-S6K1 together. The resultant IF intensity from the combined labeling of the two antibodies clearly showed efficacy of administrating everolimus.


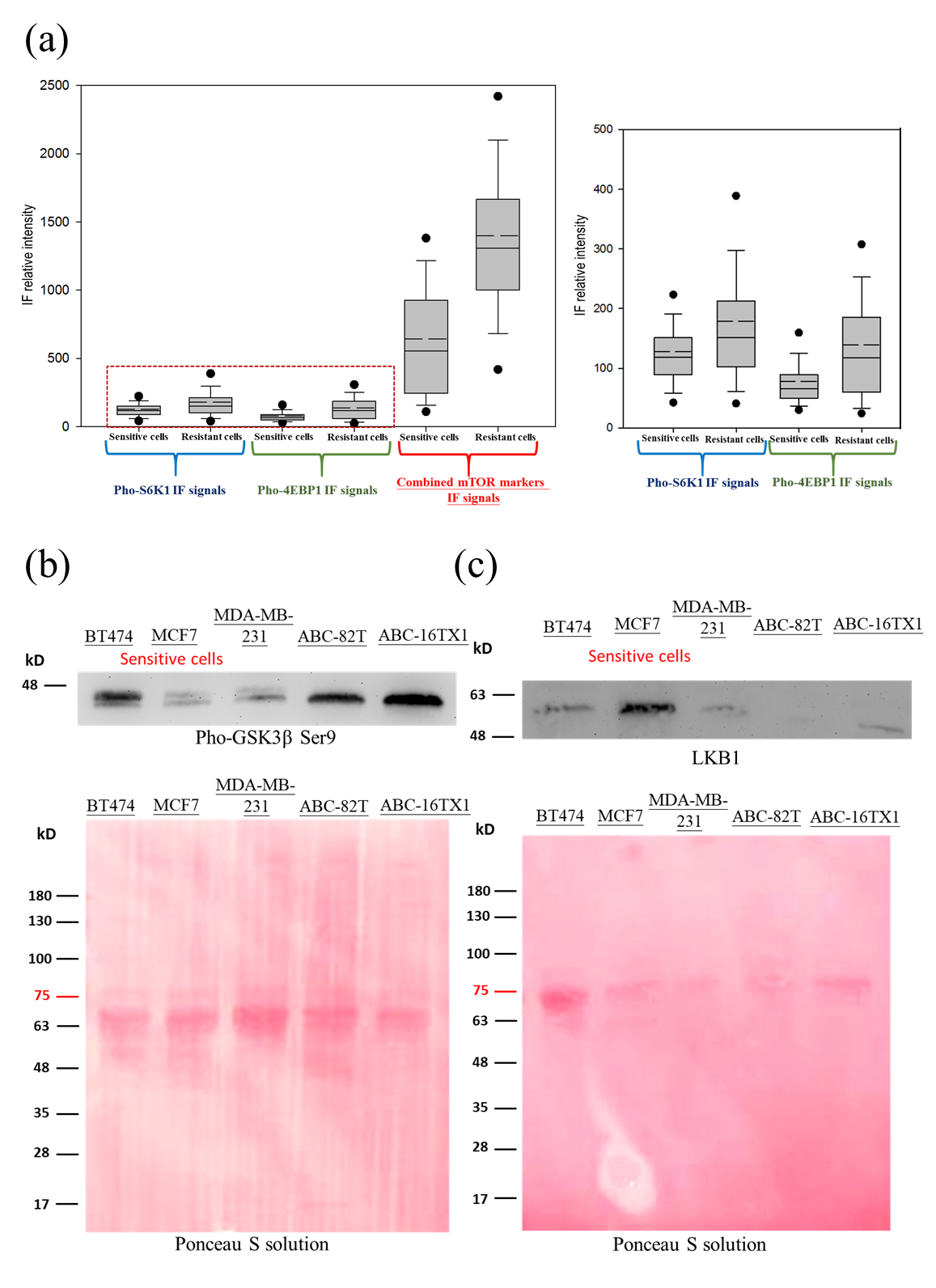


Supplementary Figure 5. (a) The two antibody-added IF intensity has higher ability to distinguish everolimus-sensitive and everolimus-resistant cells group than pho-4EBP1 and pho-S6K1 individually. (Amount of cells used in the test: 11092 cells, including six types of cell lines and PDCC.) (b) Western blot expression with ponceau S solution showing the total protein. The expression of black band at 46 kDa in western blot represents the amount of pho-GSK3β protein. (c) Western bolt expression of LKB1 for cell lines. The expression of black band at 54 kDa in western blot represents the amount of target protein. Results shows that everolimus-sensitive cells have higher LKB1 IF intensity than everolimus-resistant cells. (see text, and full-length blots are also included in a Supplementary Figure 6- Supplementary Figure 8).


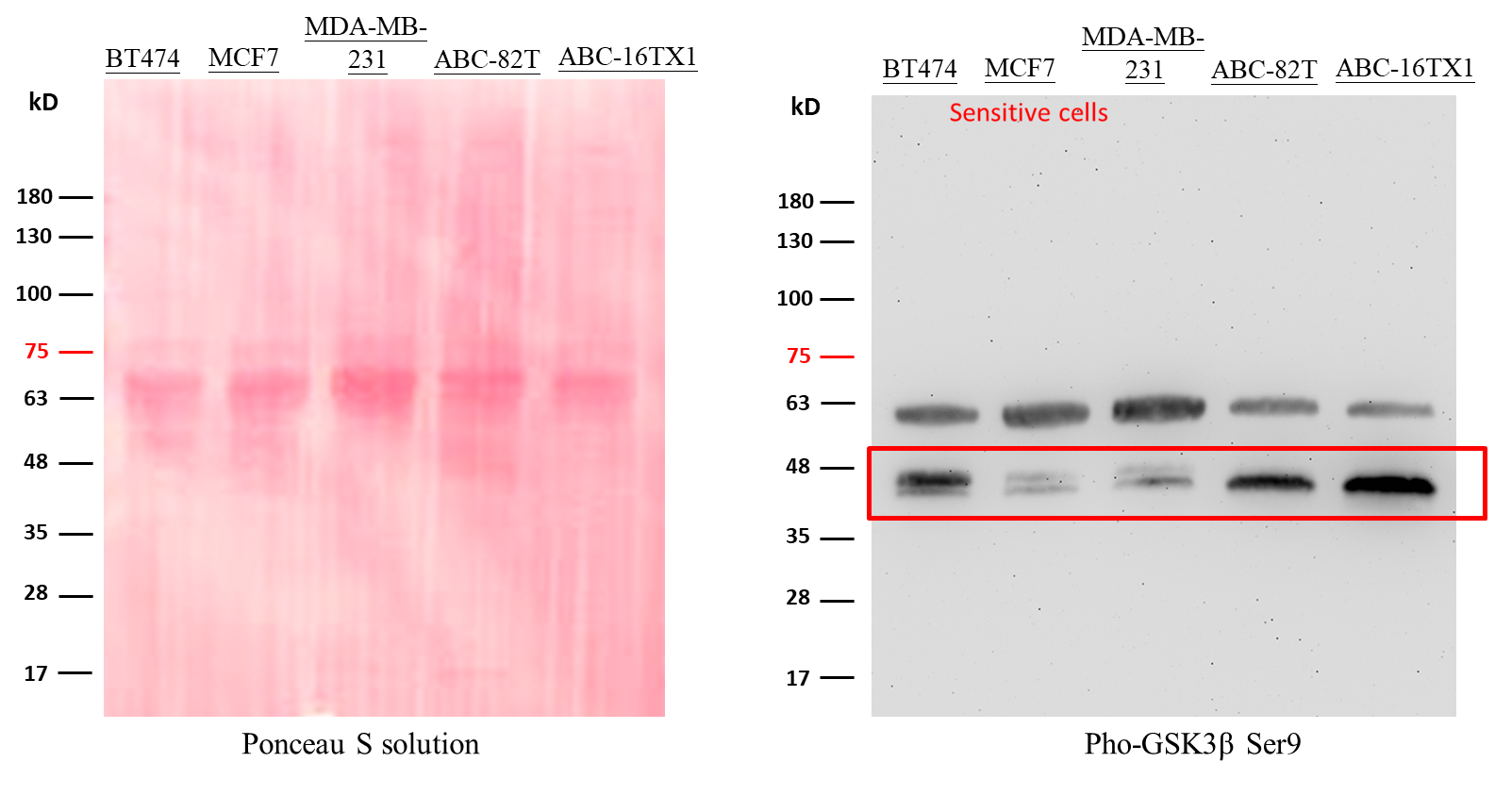


Supplementary Figure 6 The full-length western blot expression for pho-GSK3β with ponceau s solution, which shows the total protein. The expression of black band at 46 kda in western blot stand for amount of targeted protein.


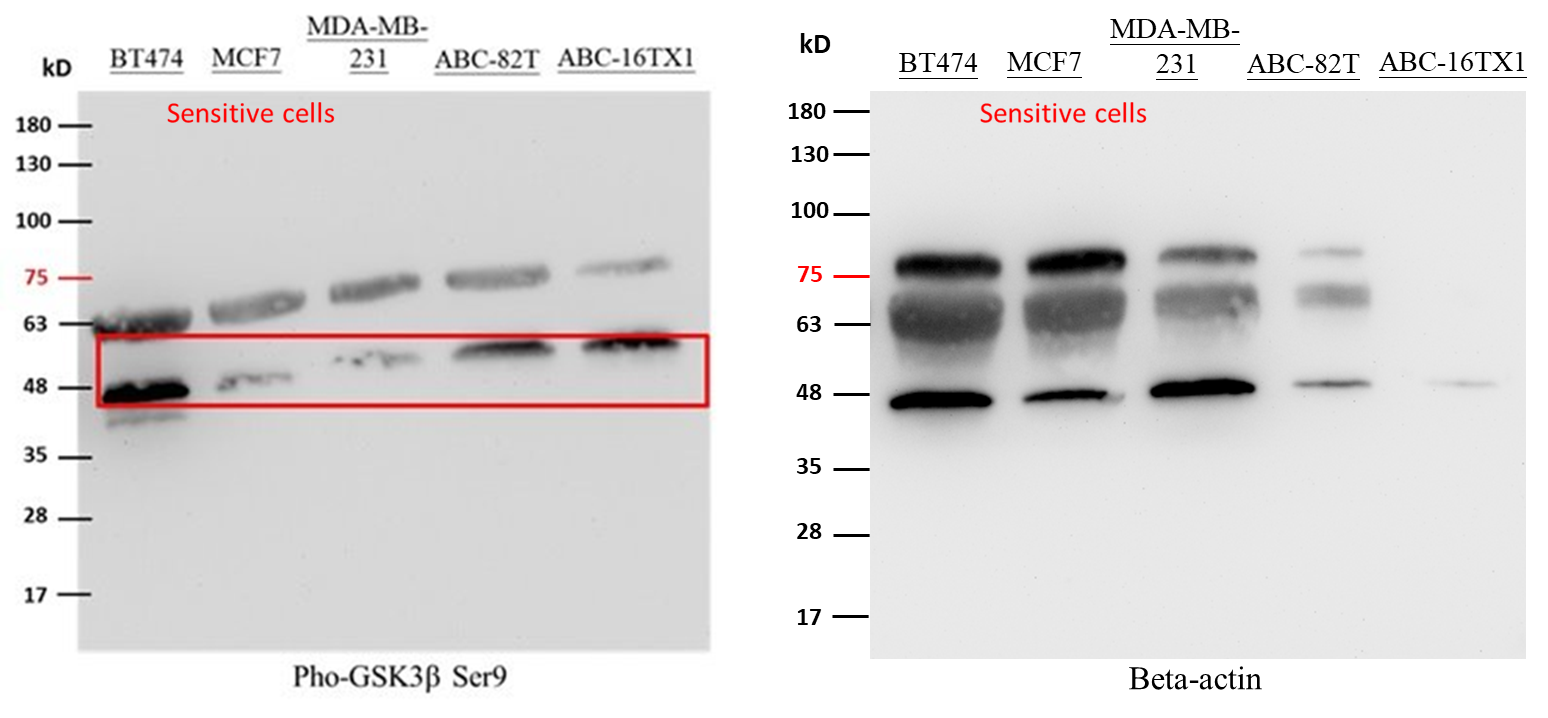


Supplementary Figure 7 The full-length western blot expressions for pho-GSK3β with beta-actin as loading control. The expression of black band at 46 kda in western blot stand for amount of pho-GSK3β, and the expressions of the black band at 48 kda represent amount of loading control.


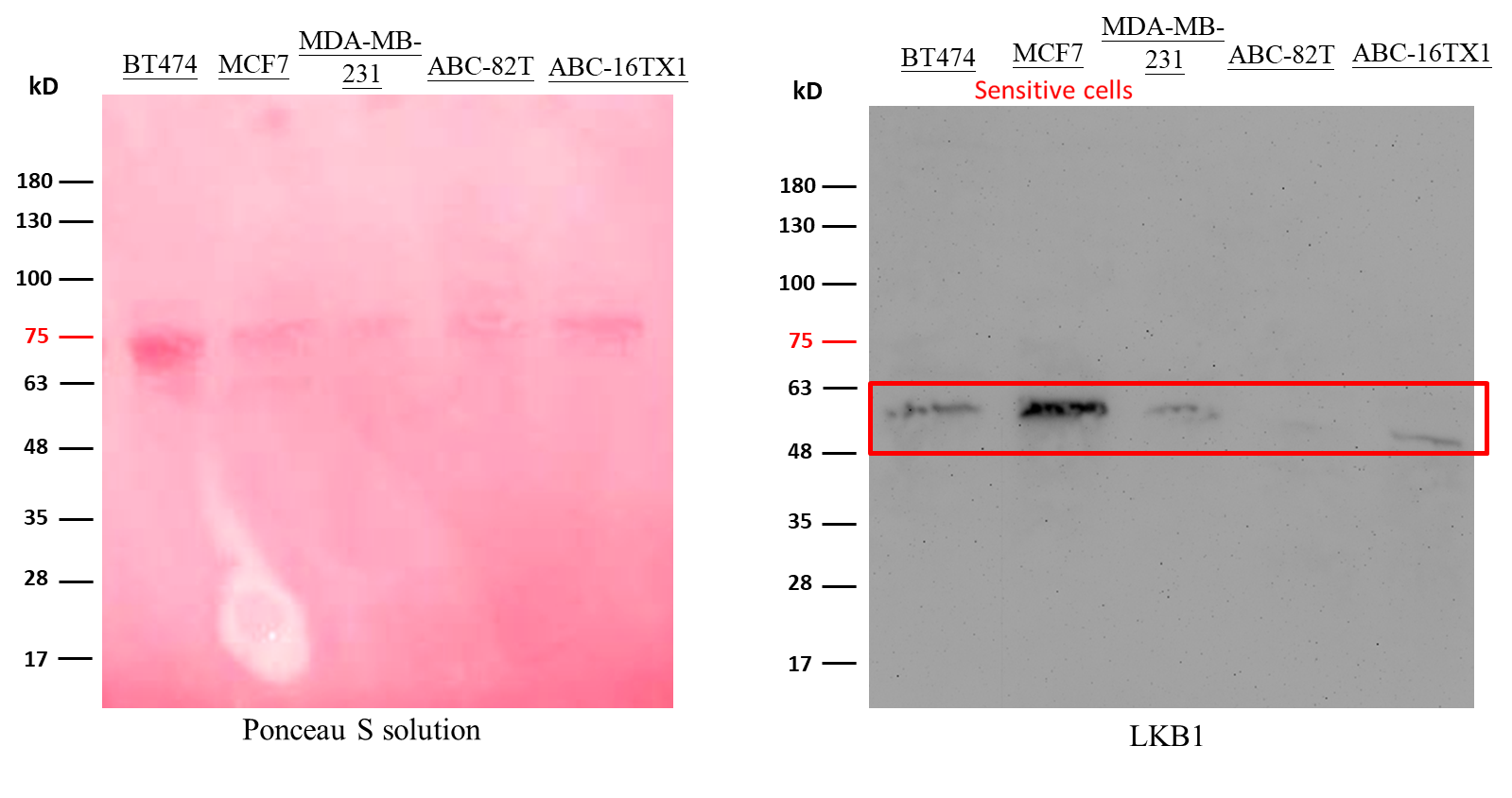


Supplementary Figure 8 The full-length western blot expression for LKB1 for cell lines with ponceau s solution, which shows the total protein. The expression of black band at 54 kda in western blot stand for amount of targeted protein.

Supplementary Table 1 Gene mutation for cell lines and PDCC used in the study [15,35-36]

|  | *p53*  *mutation* | *PI3CA mutation* | *PTEN mutation* | *KRAS mutation* |
| --- | --- | --- | --- | --- |
| *Hs578T* | Yes | None | None | Yes |
| *BT474* | Yes | Yes  (K111N) | None | None |
| *MCF7* | None | Yes  (E545K) | None | None |
| *MDA-MB-231* | Yes | None | None | Yes  (G13D) |
| *ABC-82T* | Yes | Yes  (E542K) | Unknown | Unknown |
| *ABC-16TX1* | Yes | Yes  (E545K) | Unknown | Unknown |

Supplementary Table 2 Characteristics for cell lines and PDCC used in the study

| *Cell lines and PDCC* | *Breast cancer subtype* | *Epithelial/Mesenchymal*  *property* | *Notes* |
| --- | --- | --- | --- |
| *Hs578T* | Triple negative | EpCAM negative  Viemntin positive |  |
| *BT474* | Luminal B | EpCAM positive  Viemntin negative |  |
| *MCF7* | Luminal A | EpCAM positive  Viemntin positive |  |
| *MDA-MB-231* | Triple negative | EpCAM positive  Viemntin +/- |  |
| *ABC-82T* | HER2 positive | EpCAM positive  Viemntin +/- | +/-: Positive but not obvious performance |
| *ABC-16TX1* | ER positive,  HER2 negative | EpCAM positive  Viemntin +/- | Stem cell property  (CD44 positive, CD24 negative) |

Supplementary Table 3 The group of cells by pho-S6K1 and pho-4EBP1 in the study

|  | *Cell lines and PDCC* | *Respone to everolimus from immunofluorescence* |
| --- | --- | --- |
| *Pho-S6K1 low, pho-4EBP1 low* | Hs578T, MCF7 | Sensitive |
| *Pho-S6K1 low, pho-4EBP1 low* | BT474 | Resistant |
| *Pho-S6K1 high, pho-4EBP1 low or*  *Pho-S6K1 low, pho-4EBP1 high* | MDA-MB-231  ABC-82T | Resistant |
| *Pho-S6K1 high, pho-4EBP1 high* | ABC-16TX1 | Strongly resistant |
