# Supplementary material for: Author Correction: Immunofluorescence can assess the efficacy of mTOR pathway therapeutic agent Everolimus in breast cancer models
Source: Sci Rep. 2020 Aug 19;10:14139. doi: 10.1038/s41598-020-68553-7 (PMC7434874; doi:10.1038/s41598-020-68553-7)
Supplement: Supplementary file 1 — Supplementary information [file 41598_2020_68553_MOESM1_ESM.pdf]

Immunofluorescence can assess the efficacy of mTOR pathway therapeutic agent Everolimus in breast cancer models (Supplementary Information)

Chun-Ting Kuo[1,*], Chen-Lin Chen[1,*], Chih-Chi Li[1], Guan-Syuan Huang[1], Wei-Yuan Ma[1], Wei-Fan Hsu[1], Ching-Hung Lin[2], Yen-Shen Lu[2,***], Andrew M. Wo[1,**]

[1] Institute of Applied Mechanics, National Taiwan University, Taipei 106, Taiwan
[2] Department of Oncology, National Taiwan University Hospital, Taipei 100, Taiwan
* These authors contributed equally to this paper.
** Corresponding author, e-mail: andrew@iam.ntu.edu.tw, phone: 886-2-3366-5656
*** Corresponding author, e-mail: yslu@ntu.edu.tw

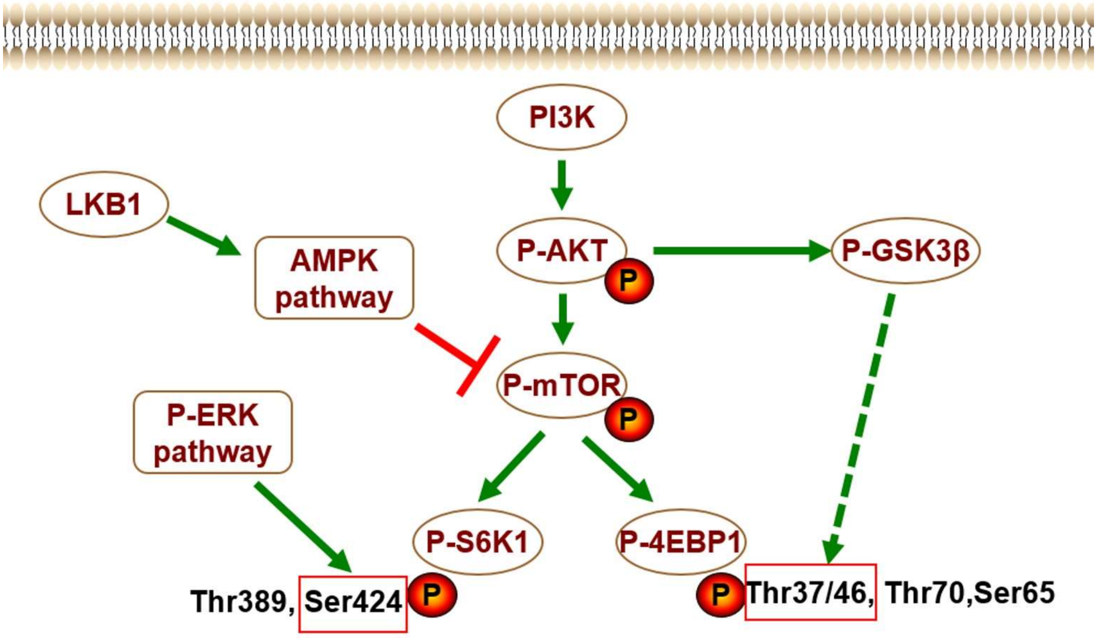

Supplementary Figure 1. Pho-4EBP1 and pho-S6K1 are not only due to AKT/mTOR pathway but activated by another target or pathway.

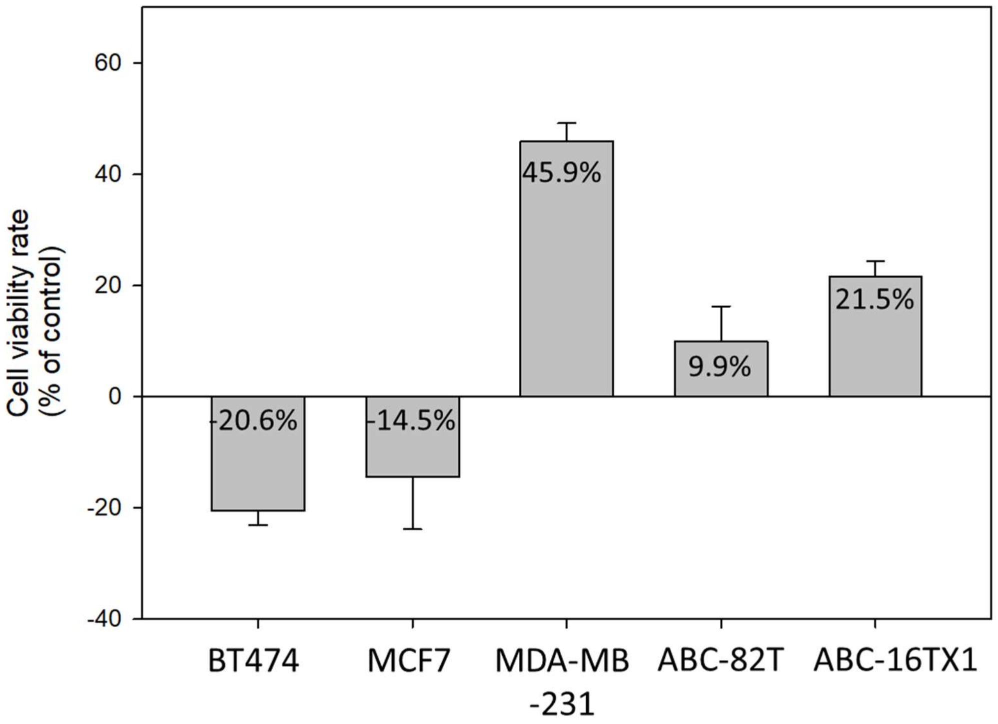

Supplementary Figure 2. Cell proliferation situations under the 200 nM everolimus treatment alone after about 24 hours. Some cells were found as sensitive to everolimus (cell growth rate is negative) where the others are resistant to everolimus (cell growth rate is positive).
(BT474, MCF7, MDA-MB-231, ABC-82T and ABC-16TX1 cells are chosen.)

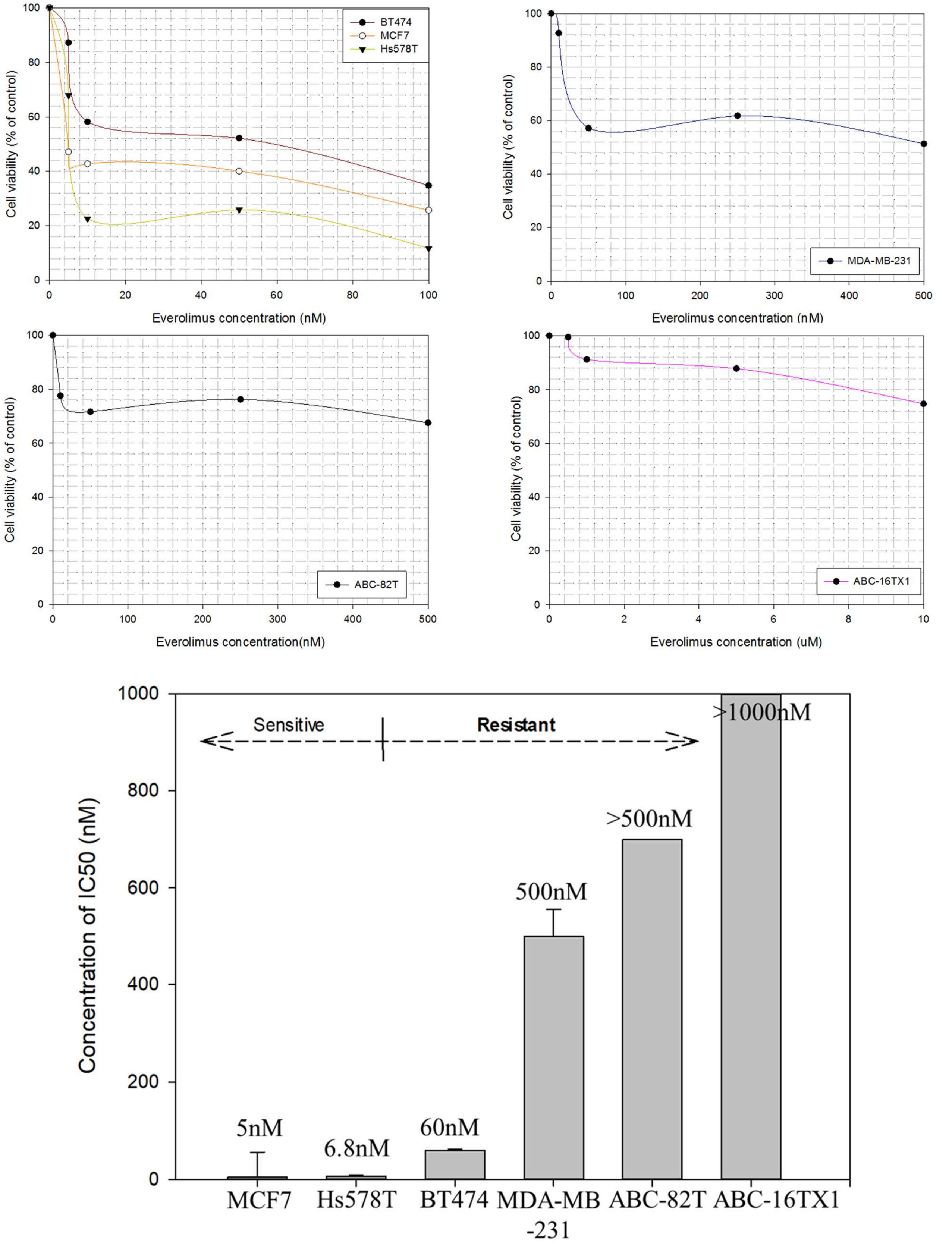

Supplementary Figure 3. Cell survival curve and concentration of IC50 to everolimus for cell lines and PDCC by MTT assay.
(Hs578T, BT474, MCF7, MDA-MB-231, ABC-82T and ABC-16TX1 cells are chosen.)

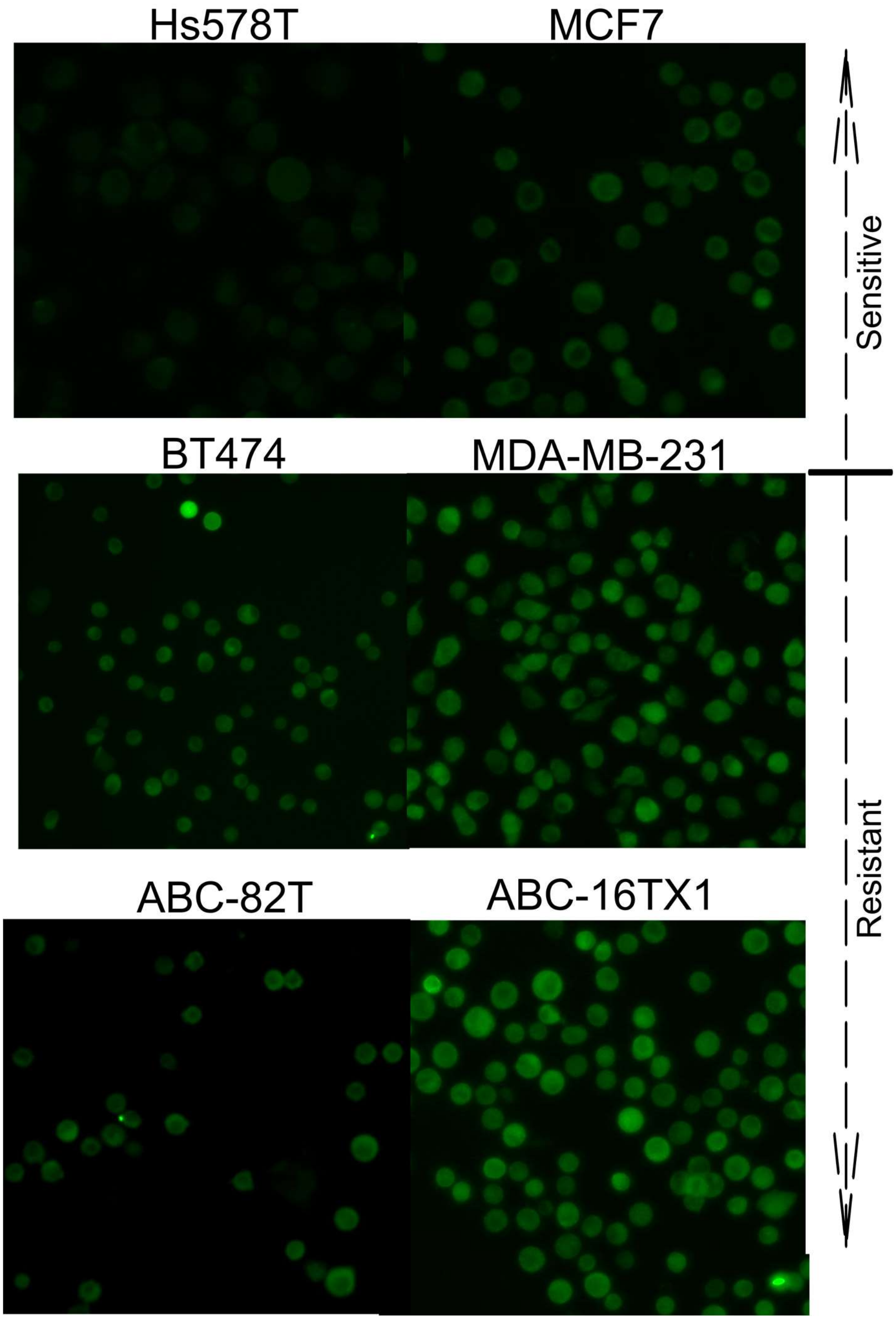

Supplementary Figure 4. Combined IF expression in six types of breast cancer cell lines and PDCC. From the experiment data, the individual use of either pho-4EBP1 or pho-S6K1 in immunofluorescence labeling might not better distinguish mTOR resistance intuitiveness than labeling both (combined) pho-4EBP1 and pho-S6K1 together. The resultant IF intensities from the combined labeling of the two antibodies clearly show efficacy of administrating everolimus.

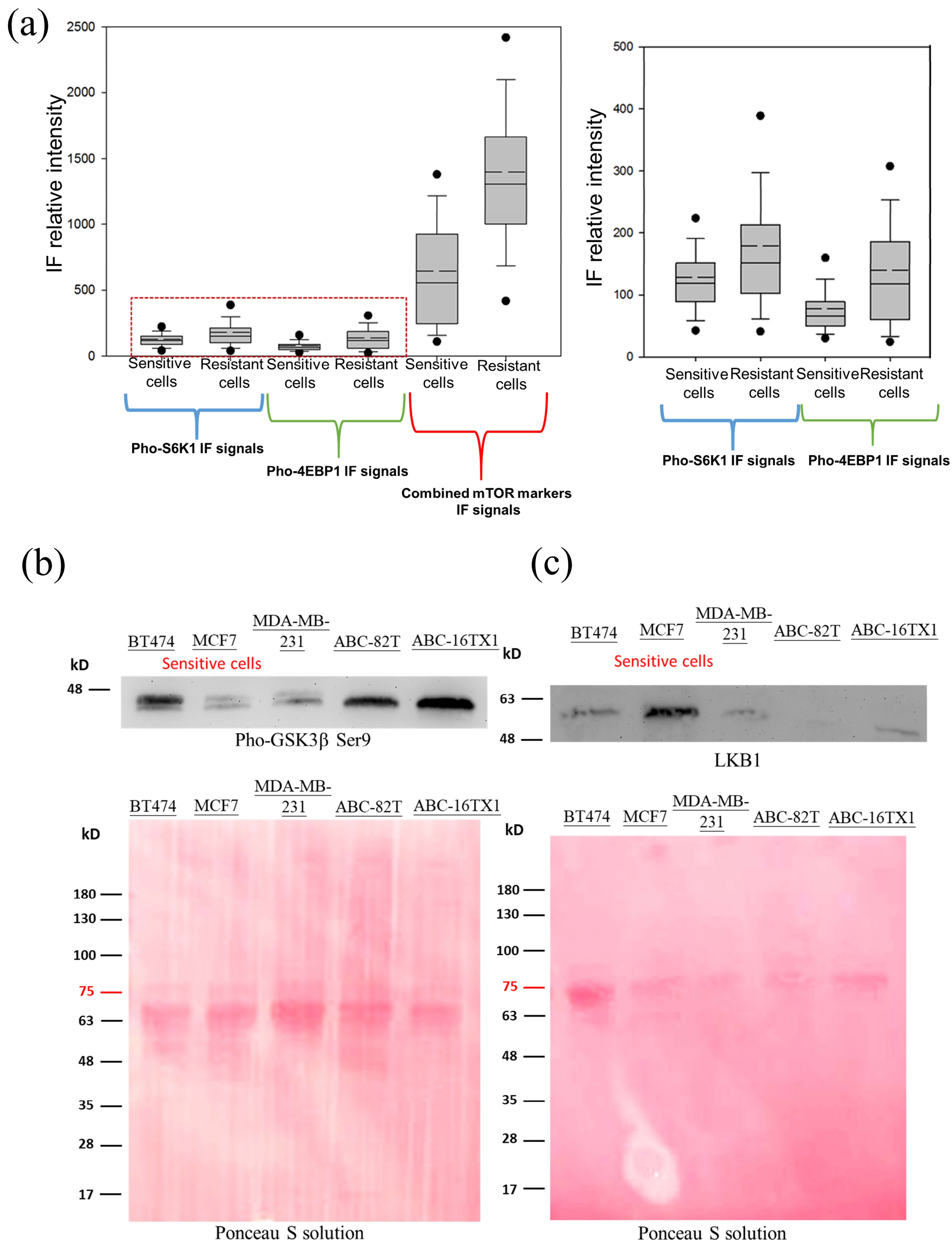
(a)
IF relative intensity
2500
2000
1500
1000
500
0
Sensitive cells
Resistant cells
Sensitive cells
Resistant cells
Sensitive cells
Resistant cells
Pho-S6K1 IF signals
Pho-4EBP1 IF signals
Combined mTOR markers IF signals
IF relative intensity
500
400
300
200
100
0
Sensitive cells
Resistant cells
Sensitive cells
Resistant cells
Pho-S6K1 IF signals
Pho-4EBP1 IF signals
(b)
BT474
MCF7
MDA-MB-231
ABC-82T
ABC-16TX1
Sensitive cells
kD
48
Pho-GSK3β Ser9
BT474
MCF7
MDA-MB-231
ABC-82T
ABC-16TX1
kD
180
130
100
75
63
48
35
28
17
Ponceau S solution
(c)
BT474
MCF7
MDA-MB-231
ABC-82T
ABC-16TX1
Sensitive cells
kD
63
48
LKB1
BT474
MCF7
MDA-MB-231
ABC-82T
ABC-16TX1
kD
180
130
100
75
63
48
35
28
17
Ponceau S solution

Supplementary Figure 5. (a) The two antibody-added IF intensity has higher ability to distinguish everolimus-sensitive and everolimus-resistant cells group than pho-4EBP1 and pho-S6K1 individually. (Amount of cells used in the test: 11092 cells, including six types of cell lines and PDCC.) (b) Western blot expressions with ponceau S solution showing the total protein. The expressions of black band at 46 kDa in western blot represent the amount of pho-GSK3β protein. (c) Western bolt expressions of LKB1 for cell lines. The expressions of black band at 54 kDa in western blot represents the amount of target protein. Results show that everolimus-sensitive cells have higher LKB1 IF intensity than everolimus-resistant cells. (see text, and full-length blots are also included in a Supplementary Figure 6- Supplementary Figure 8).

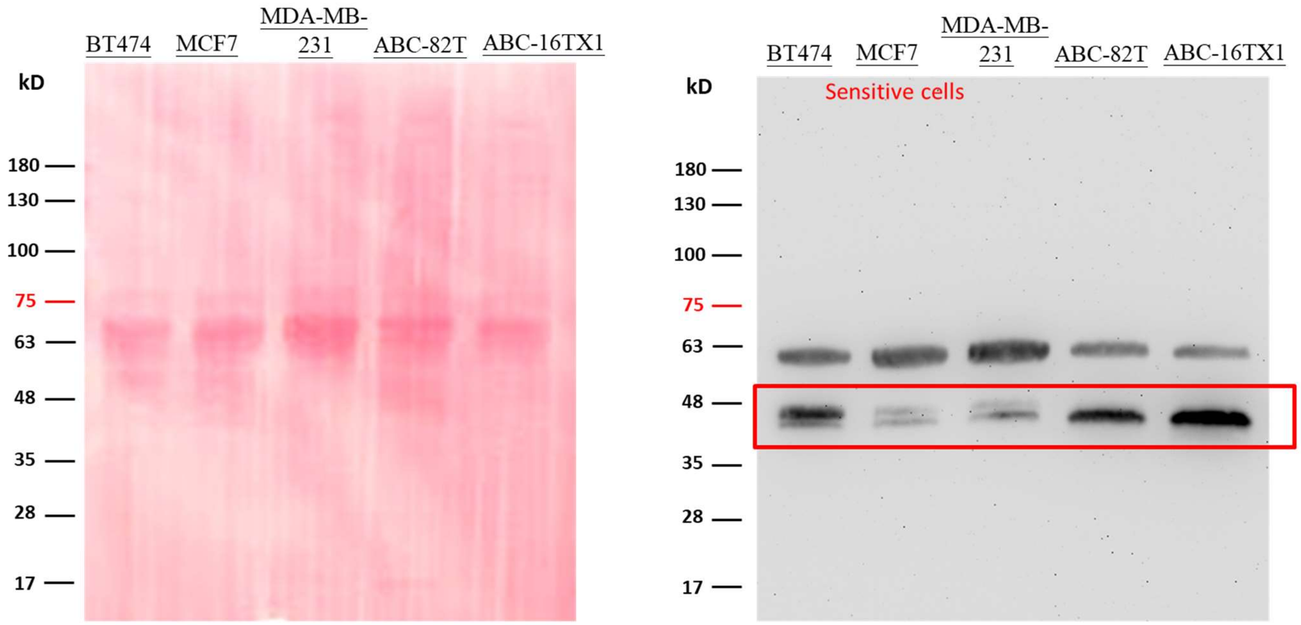

Supplementary Figure 6. The full-length western blot expressions for pho-GSK3β with ponceau s solution, which shows the total protein. The expressions of black band at 46 kda in western blot stand for amount of targeted protein.

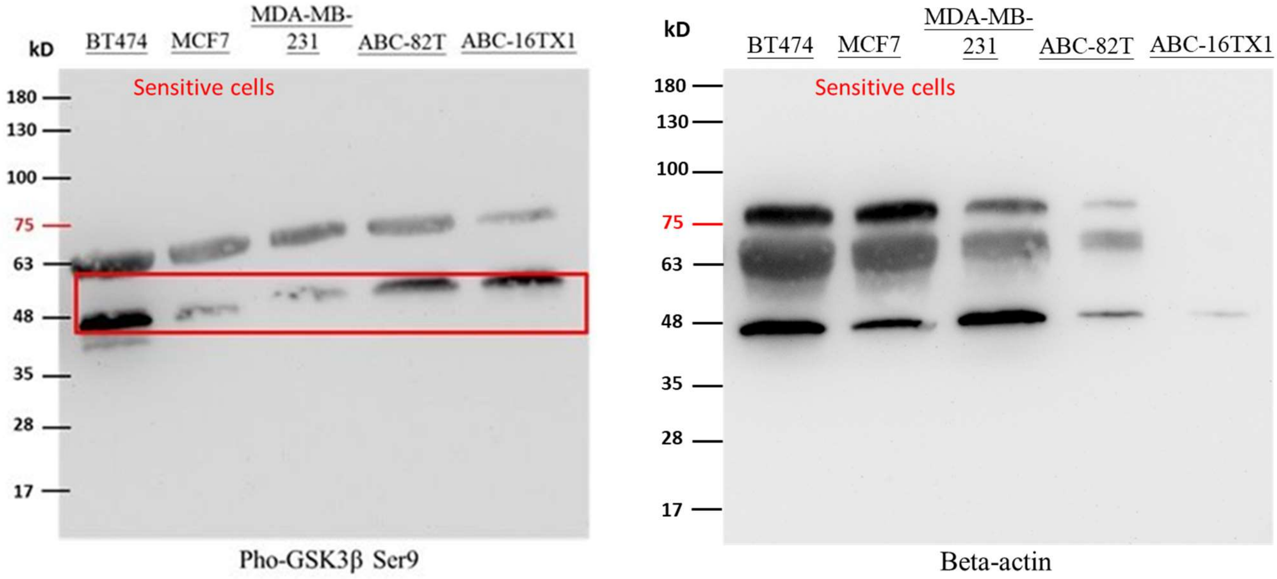

Supplementary Figure 7. The full-length western blot expressions for pho-GSK3β with beta-actin as loading control. The expressions of black band at 46 kda in western blot stand for amount of pho-GSK3β, and the expressions of the black band at 48 kda represent amount of loading control.

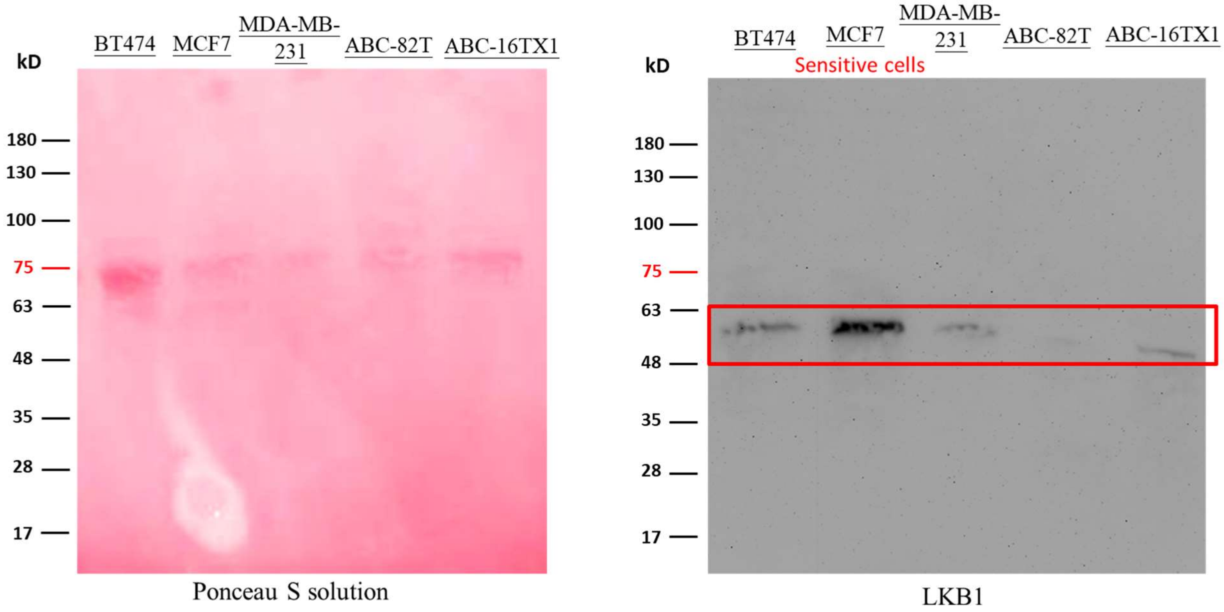

Supplementary Figure 8. The full-length western blot expressions for LKB1 for cell lines with ponceau s solution, which shows the total protein. The expressions of black band at 54 kda in western blot stand for amount of targeted protein.

Supplementary Table 1. Gene mutation for cell lines and PDCC used in the study [25,38-39]

	p53 mutation	*PI3CA mutation*	*PTEN mutation*	*KRAS mutation*
Hs578T	Yes	None	None	Yes
BT474	Yes	Yes (K111N)	None	None
MCF7	None	Yes (E545K)	None	None
MDA-MB-231	Yes	None	None	Yes (G13D)
ABC-82T	Yes	Yes (E542K)	Unknown	Unknown
ABC-16TX1	Yes	Yes (E545K)	Unknown	Unknown

Supplementary Table 2. Characteristics for cell lines and PDCC used in the study

Cell lines and PDCC	*Breast cancer subtype*	*Epithelial/Mesenchymal property*	*Notes*
Hs578T	Triple negative	EpCAM negative<br>Vimentin positive	
BT474	Luminal B	EpCAM positive<br>Vimentin negative	
MCF7	Luminal A	EpCAM positive<br>Vimentin positive	
MDA-MB-231	Triple negative	EpCAM positive<br>Vimentin +/-	
ABC-82T	HER2 positive	EpCAM positive<br>Vimentin +/-	+/-: Positive but not obvious performance
ABC-16TX1	ER positive,<br>HER2 negative	EpCAM positive<br>Vimentin +/-	Stem cell property<br>(CD44 positive, CD24 negative)

Supplementary Table 3. Four groups of cells by pho-S6K1 and pho-4EBP1 IF intensities in the study

Group	*Cell lines and PDCC*	*IF intensities*	*Response to everolimus from immunofluorescence*
1	Hs578T, MCF7	*Pho-S6K1 low, pho-4EBP1 low*	Sensitive
2	BT474	*Pho-S6K1 low, pho-4EBP1 low*	Resistant
3	MDA-MB-231<br>ABC-82T	*Pho-S6K1 high, pho-4EBP1 low or Pho-S6K1 low, pho-4EBP1 high*	Resistant
4	ABC-16TX1	*Pho-S6K1 high, pho-4EBP1 high*	Strongly resistant
